# High‐Resolution In‐Situ Synchrotron X‐Ray Studies of Inorganic Perovskite CsPbBr_3_: New Symmetry Assignments and Structural Phase Transitions

**DOI:** 10.1002/advs.202003046

**Published:** 2021-07-11

**Authors:** Sizhan Liu, Alexander R. DeFilippo, Mahalingam Balasubramanian, Zhenxian Liu, SuYin Grass Wang, Yu‐Sheng Chen, Stella Chariton, Vitali Prakapenka, Xiangpeng Luo, Liuyan Zhao, Jovan San Martin, Yixiong Lin, Yong Yan, Sanjit K. Ghose, Trevor A. Tyson

**Affiliations:** ^1^ Department of Physics New Jersey Institute of Technology Newark NJ 07102 USA; ^2^ Advanced Photon Source Argonne National Laboratory Argonne IL 60439 USA; ^3^ Department of Physics University of Illinois at Chicago Chicago IL 60607‐7059 USA; ^4^ Center for Advanced Radiation Sources University of Chicago Argonne IL 60439 USA; ^5^ Department of Physics University of Michigan Ann Arbor MI 48109‐1040 USA; ^6^ Department of Chemistry and Biochemistry San Diego State University San Diego CA 92182 USA; ^7^ National Synchrotron Light Source II Brookhaven National Laboratory Upton NY 11973 USA

**Keywords:** all‐inorganic perovskites, CsPbBr_3_, in‐situ single‐crystal diffraction, local structure, phase transitions, space groups

## Abstract

Perovskite photovoltaic ABX_3_ systems are being studied due to their high energy‐conversion efficiencies with current emphasis placed on pure inorganic systems. In this work, synchrotron single‐crystal diffraction measurements combined with second harmonic generation measurements reveal the absence of inversion symmetry below room temperature in CsPbBr_3_. Local structural analysis by pair distribution function and X‐ray absorption fine structure methods are performed to ascertain the local ordering, atomic pair correlations, and phase evolution in a broad range of temperatures. The currently accepted space group assignments for CsPbBr_3_ are found to be incorrect in a manner that profoundly impacts physical properties. New assignments are obtained for the bulk structure: $Im\bar{3}$ (above ≈410 K), *P*2_1_/*m* (between ≈300 K and ≈410 K), and the polar group *Pm* (below ≈300 K), respectively. The newly observed structural distortions exist in the bulk structure consistent with the expectation of previous photoluminescence and Raman measurements. High‐pressure measurements reveal multiple low‐pressure phases, one of which exists as a metastable phase at ambient pressure. This work should help guide research in the perovskite photovoltaic community to better control the structure under operational conditions and further improve transport and optical properties.

## Graphical abstract

Examination of single‐crystal X‐ray diffraction images between ≈500 and ≈100 K yields new symmetry assignments for CsPbBr_3_. The space groups are: Im‐3 above ≈410 K, P2_1_/m between ≈410 K and ≈300 K, and Pm below ≈300 K. A unit cell volume of ≈2a_p_ × 2a_p_ × 2a_p_ is maintained. Local structural measurements reveal non‐centrosymmetric short‐range order above 300 K.

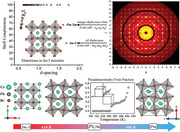

1

Hybrid perovskite photovoltaic ABX_3_ systems have been systematically studied for the past decades due to their high energy‐conversion efficiencies (in excess of ≈25% ^[^ ^1^, ^2^, ^3^ ^]^). In these systems, the A site is occupied by an organic cation, the B site is occupied by Pb, and the X site is occupied by halides (Cl, Br, or I). The organic component at the A site with charge +1 is believed to be the Achilles' heel accounting for the degradation of the hybrid perovskite system at high temperature and in the presence of moisture. Efforts to mitigate this weakness of the hybrid systems are being aggressively pursued. All‐inorganic analogs of the hybrid system with the A site occupied by alkali atoms, such as Cs or Rb, are being investigated.^[^ ^4^, ^5^, ^6^, ^7^, ^8^ ^]^ These systems have high stability and high open‐circuit voltages. Of particular interest is CsPbBr_3_ which is known to have a high carrier mobility and a large carrier diffusion length.^[^ ^9^, ^10^, ^11^ ^]^ Understanding the basic physical principles underlying the exceptional properties under operational conditions of this material requires a detailed determination of the crystal structure.

Early experiments by Moller^[^ ^12^, ^13^ ^]^ and Cola et al.^[^ ^14^ ^]^ found a monoclinic cell at room temperature while Hirotsu et al.^[^ ^15^ ^]^ assigned a *Pnma* orthorhombic cell based on the neutron diffraction method. Specifically, Hirotsu et al. observed systematic absence violations and superlattice reflections in this orthorhombic cell but excluded them due to their weak intensities. The superlattice reflection intensities were found to exhibit changes at the bulk transition temperatures indicating that the corresponding planes are an integral part of the CsPbBr_3_ crystal lattice.^[^ ^15^ ^]^

In the most recent assessments focusing on photovoltaic properties, the crystal structure of CsPbBr_3_ was studied mainly by powder diffraction and laboratory diffraction methods.^[^ ^9^, ^10^, ^11^, ^16^ ^]^ utilizing the early assigned space groups by Hirotsu etal. These methods are known to have difficulty in detecting very weak reflections which may lead to the assignment of wrong space groups. The early work suggested a second‐order change from an orthorhombic to a tetragonal space group at 361 K with increasing temperature, indicating a *Pnma* (#62, centrosymmetric) to *P*4/*mbm* (#127, centrosymmetric) space group change. At 403 K, a first‐order transition from *P*4/*mbm* to the $Pm\bar{3}m$ (#221, centrosymmetric) space group was found. It is noted that all of these space groups are centrosymmetric. In terms of the simple perovskite cell with edge‐length *a*_p_ ≈ 5.8 Å, these transitions correspond to changes in the unit cell volume from $\sqrt{2}a_{p}\times \sqrt{2}a_{p}\times 2a_{p}$ to $\sqrt{2}a_{p}\times \sqrt{2}a_{p}\times a_{p}$, and from $\sqrt{2}a_{p}\times \sqrt{2}a_{p}\times a_{p}$ to *a*_p_ × *a*_p_ × *a*_p_, respectively, as the temperature is increased. Based on the early symmetry assignments, the results were refined more recently in powder diffraction measurements,^[^ ^17^ ^]^ nuclear magnetic resonance, and nuclear quadrupole resonance studies.^[^ ^18^ ^]^ However, no independent structural symmetry assignments were performed on single crystals to determine the space group symmetries unambiguously. At the level needed for accurate theoretical models for electron transport, thermal properties, and to develop accurate atomic potentials, these details have been lacking in the literature.

Recent theoretical and experimental physical property determinations have been found to be inconsistent with the established space groups. Density functional theory (DFT) based lattice dynamics calculations exploring the phonon stable models of ABX_3_ inorganic alkali halide systems at low temperature reveal instabilities in the current cubic, tetragonal, and orthorhombic phase of CsPbBr_3_.^[^ ^19^ ^]^ The stable phase is predicted to be monoclinic *P*2_1_/*m*. In the specific class of Pb‐based alkali halides APbX_3_, DFT modeling reveals distortions as classic double‐well potentials relative to the cubic phase.^[^ ^20^ ^]^ The results indicate collective ferroelectric polarization which may be observable on the nanoscale. In quite recent experiments, quantum dots of CsPbBr_3_ (cubic‐shaped nano‐crystals with ≈5 nm edge lengths) were found to exhibit a finite electric polarization. The value of the saturation polarization at 298 and 77 K were found to be ≈0.018 µC cm^−^^2^ and ≈0.25 µC cm^−^^2^, respectively.^[^ ^21^ ^]^ A significant piezoelectric response has been found in films of this material (≈40 nm to ≈260 nm thick).^[^ ^22^ ^]^ Room temperature remnant polarization values up to ≈0.03 µC cm^−^^2^ were observed. These recent studies suggest the existence of non‐centrosymmetric structures and new symmetry assignments in bulk CsPbBr_3_ at room temperature and below. In the work presented here, independent structural measurements were conducted on micron‐scale as‐grown single crystals to assess the structure of this material on multiple length scales.

2

To determine the appropriate symmetries and phase transitions in this material, high‐quality single crystals (orange phase) were synthesized and studied. An as‐grown single crystal with ≈50 µm cube edges was utilized for single‐crystal diffraction measurements. Second harmonic generation (SHG) measurements were conducted to assess the nature of the crystal symmetry below room temperature. Differential scanning calorimetry (DSC) measurements between 300 and 700 K indicate that both transitions in this region are first‐order with the transition near 350 K having a smaller ΔH, relative to that at ≈410 K. Single‐crystal X‐ray diffraction (XRD) measurements were conducted between 100 and 450 K, utilizing a detector with a high dynamic range to detect both weak and strong reflections.

Our high‐resolution synchrotron‐based single‐crystal diffraction measurements reveal that between ≈450 and 410 K, the space group is cubic $Im\bar{3}$ (#204, centrosymmetric). It undergoes a first‐order cubic‐to‐monoclinic phase transition at ≈410 K. Between ≈410 and 300 K, the *P*2_1_/*m* (#11, centrosymmetric) monoclinic space group is maintained. A first‐order isostructural transition within the *P*2_1_/*m* space group is observed at ≈350 K. As temperature goes below ≈300 K, a second‐order phase transformation is identified. Below 300 K, the weak superlattice peaks which appear below ≈410 become enhanced in the monoclinic *Pm* (#6, polar) space group. SHG measurements confirmed the absence of inversion symmetry below 300 K. The currently accepted space groups are found to be incorrect in a manner that profoundly impacts physical properties. The unit cell dimensions, 2*a*_p_ × 2*a*_p_ × 2*a*_p_, are preserved in the studied temperature range (450 K and below). The previously reported space groups in the literature can be recovered if exclusively the dominant reflections are utilized in structural analysis. Raman scattering measurements between 100 and 830 K and pair distribution function measurements between 10 and 500 K indicate the presence of an isostructural order‐disorder transition near 170 K. High‐pressure measurements between 1 atm and 13 GPa indicate the resilience of the material under pressure and reveal a first‐order phase transition at ≈1 GPa, and successive continuous transitions near 2, 6, and 13 GPa. Low‐pressure (≈1 GPa) room temperature measurements recover the low‐temperature properties. Theoretical models of these materials will be more heavily constrained by the utilization of the high‐resolution structural data over the broad range of temperatures and pressures provided in this work. The transitions at ≈350 and ≈410 K are found to be first‐order. The first‐order nature of these successive transitions will impact transport properties if materials are cooled rapidly once heated significantly above room temperature. The details of the experimental and modeling methods are given in the Supporting Information. Crystallographic data on the specific phases at representative temperatures are also provided.

Differential scanning calorimetric measurements on CsPbBr_3_ crystals were used to identify the nature of the observed structural phase transitions. Figure S1, Supporting Information, shows the heating and warming curves with transitions evident near 362 and 402 K. These measurements reveal hysteretic behavior covering the region between 360 and 365 K and a large peak near the transition at 402 K (with cooling/warming offset), indicating that both transitions are first‐order with the lower transition revealing a significantly lower Δ*H* in the lower‐temperature transition. The weakness of the peak near 360 K in earlier measurements^[^ ^16^ ^]^ led to its assignment as a second‐order transition. As seen below, our X‐ray diffraction measurements on single‐crystal CsPbBr_3_ confirmed the nature of both phase transitions as first‐order. Discontinuities are seen in the temperature dependence of the lattice parameters and/or Bragg peak intensities at these transitions.

To understand the changes in structure over a broad temperature range, temperature‐dependent Raman spectroscopy measurements on single‐crystal CsPbBr_3_ were carried out. The low‐energy spectra for limited temperatures have been previously reported.^[^ ^23^ ^]^ In this work, we present a more complete temperature range to enable the identification of phase transitions. The full Raman spectra can be found in Figure S2, Supporting Information. Spectra are systematically shifted in intensity for clarity. **Figure** **1**a shows a contour plot of the temperature dependence of the unpolarized Raman spectra from 100 to 500 K, indicating four transitions as horizontal lines at ≈170, 300, 350, and 410 K. In particular, the 73 and 79 cm^−1^ modes soften as temperature increases and merge to become a single peak at ≈300 K revealing a second‐order phase transition. Note the abrupt changes in the position of these peaks near 350 K. Furthermore, these peaks undergo significant broadening above 410 K. These two modes deserve a detailed analysis due to their significant variation with temperature. Hence, the spectra are fitted to a sum of Lorentzian functions. As shown in Figure 1b, the black open circles represent the experimental data, the solid red line represents the total fit, and the dashed lines show the individual Lorentzian components. A double‐peak structure is shaded in red and blue for the modes at 73 and 79 cm^−1^, respectively.

**Figure 1:**
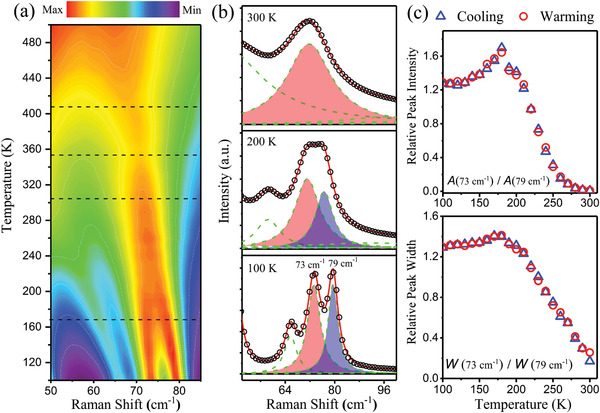
a) Contour plot of the temperature dependence of the Raman spectra. b) Low energy region of Raman spectra fitted by a sum of Lorentzian functions. The black open circles give the experimental data, the solid red line represents the sum of the fitting functions, and the dashed lines show the individual fitting components. A double‐peak structure is shown, which is shaded in red and blue, for the modes at 73 and 79 cm^−1^, respectively. The ratio of fitted peak area and peak width are shown in panel (c), indicating a phase transitions onset near 170 and 300 K.

Figure 1c shows the temperature dependence of the ratio of the fitted peak areas and peak widths for this double‐peak structure. With increasing temperature, the Raman scattering intensity ratio of the high‐energy to the low‐energy peak grows substantially between 100 and 170 K, while showing a kink at ≈170 K. This is followed by a strong decrease of 79 cm^−1^ mode intensity as temperature increases. The peak area ratio vanishes near 300 K. We note that the cooling and warming curves coincide, indicating no hysteresis. Hence, the phase transition at 170 K is second‐order. These transitions near 170 and 300 K were not previously identified in structural measurements.

DFT simulations indicate that these specific modes involve complex motion of the Cs and Br atoms in which layers of Cs atom exhibit shear‐type motion triggered by asymmetric distortion of the PbBr_6_ polyhedra. Table S1, Supporting Information, gives the DFT derived phonon frequencies for the orthorhombic cell while the atomic displacements of representative modes at 32, 56, 73, 79, 137, and 158 cm^−1^ are displayed in Figure S3b, Supporting Information. We note that, in the case of CsPbCl_3_, the Raman measurements reveal an order–disorder transition near ≈170 K.^[^ ^24^ ^]^ Very early nuclear quadrupole magnetic resonance measurements on CsPbBr_3_ reveal the appearance of an additional line near 167 K, which broadens and disappears for higher temperatures indicating that it is second‐order in nature.^[^ ^25^ ^]^ Pair distribution function (PDF) measurements will be used to explore the nature of the transition near 170 K and the higher temperature transitions. Assessing the space groups and structural symmetry requires detailed high‐resolution single‐crystal XRD measurements.

Accurate structural parameters of bulk CsPbBr_3_ were derived from detailed synchrotron‐based single‐crystal diffraction measurements between 100 and 450 K on warming using an as‐grown cube‐shaped single crystal of edge ≈50 µm. To observe both strong and weak reflections simultaneously, a detector with a large dynamic range was utilized. Full single‐crystal data sets (no symmetry assumptions) were collected in 10 K steps over the temperature range. Systematic exploration of the space groups which best fit the data and account for all observed reflections were conducted.

As indicated above, earlier experimental studies^[^ ^15^, ^16^, ^17^ ^]^ claimed that the high‐temperature phase of CsPbBr_3_ is cubic *Pm*‐3*m* structure with a lattice parameter *a*_p_ ≈ 5.87 Å. However, by carefully examining the reciprocal lattice images (for temperatures up to 450 K) obtained from high‐resolution single‐crystal X‐ray diffraction data, half‐integers (*hkl*) peaks are observed which reveals that the lattice parameters and space groups should be revised. By not accounting for these critical weak reflections, the previously reported models can not properly characterize the properties in this system. We note that although no symmetry assignments were made utilizing all reflections as done here, the presence of these half‐integer reflections was reported in early work.^[^ ^12^, ^13^, ^14^, ^15^ ^]^

In this work, the single‐crystal diffraction derived structures are present in **Figure** **2**a. The space group above 410 K is found to be cubic $Im\bar{3}$ with unit cell volume, 2*a*_p_ × 2*a*_p_ × 2*a*_p_. Only very weak rotation and tilting can be observed in the cubic structure. Below 410 K, the cubic‐to‐monoclinic phase transition is characterized by the motions of Br and Cs atoms off high‐symmetry positions in the unit cell yielding space group *P*2_1_/*m* with cell volume, 2*a*_p_ × 2*a*_p_ × 2*a*_p_. Below 300 K, the structure is in the monoclinic *Pm* space group. The changes in temperature‐dependent lattice parameters also reveal the phase transitions shown in Figures 2d,e.

**Figure 2:**
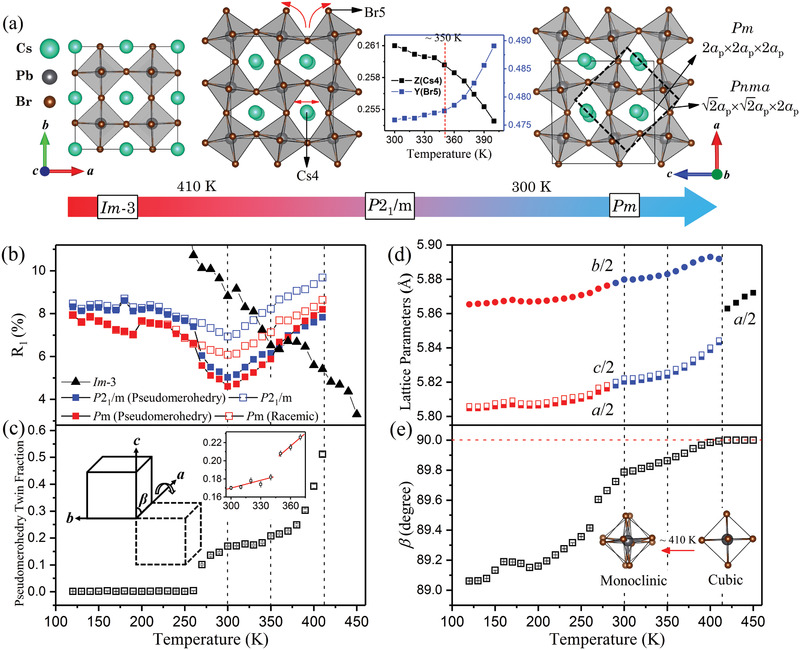
a) The temperature‐dependent structure of CsPbBr_3_ from single‐crystal synchrotron X‐ray diffraction measurements. Above 410 K, the space group is cubic $Im\bar{3}$. Between 410 and 300 K, the structure is monoclinic *P*2_1_/*m*. The inset shows the Z fractional coordinate of Cs4 and the Y fractional coordinate of Br5 as a function of temperature. An isostructural phase transition is observed at 350 K. Below 300 K, the space group is *Pm*. The *Pm* and previously reported *Pnma* unit cells are given as the solid and dotted lines, respectively. b) The quality‐of‐fit parameter, *R*_1_, of *Pm*, *P*2_1_/*m*, and *I*‐3*m* structures. The solid squares indicate the *R*1 parameters which incorporate a pseudomerohedry twin law matrix (1 0 0, 0 −1 0, 0 0 −1), while the open squares are the *R*1 parameters of racemic twinning for *Pm* structure and no twinning for *P*2_1_/*m* structure. The temperature‐dependent pseudomerohedry twin domain fraction is shown in (c). The single‐crystal diffraction derived lattice parameters as a function of temperature are given in (d) and (e).

It should be emphasized that reciprocal space images were examined in detail for all regions of temperature to assign the appropriate space groups. **Figure** **3**a shows the single‐crystal XRD reciprocal lattice image of the (*hk* 0) plane at 450 K. The (*hkl*) grid corresponds to the previously reported $Pm\bar{3}m$ unit cell with lattice constant *a*_p_ ≈ 5.87 Å. The insets show the 3D intensity of some selected reflections with their asymmetric diffuse scattering background evident. The observation of half‐integer *h* or *k* values indicates the corresponding lattice constants should be doubled. To systematically compare data for the whole temperature range, the reciprocal lattice precession images for each temperature were calculated based on the same unit cell dimension, *a*_p_ × *a*_p_ × *a*_p_. The temperature‐dependent intensity map of (*h* ‐2 0) and (*h* ‐2.5 0) reflections are given in Figure 3b,c, respectively, which are the selected regions shown in panel (a). The temperature‐dependent structural distortion and symmetry changes can be characterized by the intensity variation of the reflections. As an example of the dominant reflections, the intensity of the (2 ‐2 0) reflection shows an abrupt change from ≈350 to 410 K. For the superlattice reflections, the intensity of (2 ‐2.5 0) reflection vanishes above ≈410 K, while the (1.5 ‐2.5 0) reflection intensity showing a kink at the same temperature. In addition to the change in slope at ≈410 K for the (2 ‐2 0) peak intensity in Figure 3b, a similar abrupt slope change indicates a transition also occurring at ≈350 K. No change in the space group is found by our structural studies leading to our assignment of an isostructural transition. This type of transition is rare, strictly first‐order in nature, and requires a high‐order expansion of the Landau free energy.^[^ ^26^, ^27^ ^]^ Below ≈410 K, the appearance of the additional reflections reveals the cubic‐to‐monoclinic phase transition.

**Figure 3:**
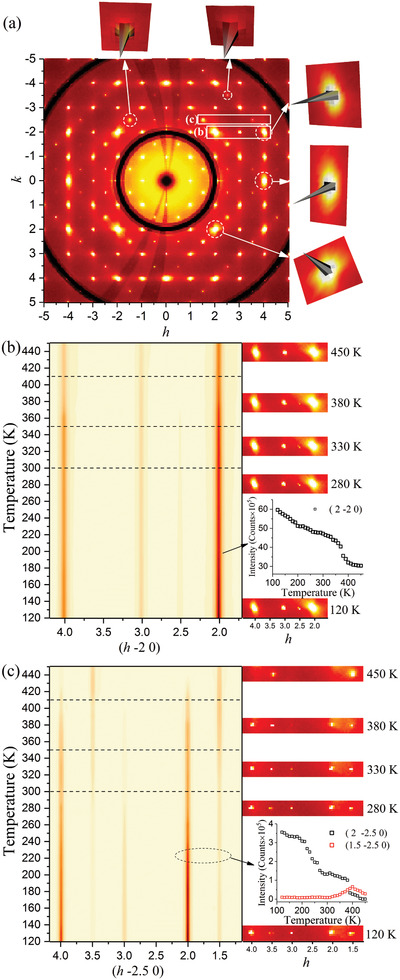
a) Single‐crystal X‐ray diffraction reciprocal lattice image of the (*hk* 0) plane at 450 K. The (*hkl*) grid corresponds to the previously reported $Pm\bar{3}m$ space group with lattice‐constant *a*_p_ ≈ 5.87 Å. The insets show the 3D intensity of some selected reflections with an asymmetric diffuse scattering background. Diffraction spots with half‐integer *h* and *k* values are observed, indicating the correct lattice constant should be doubled. The temperature‐dependent intensity of (*h* −2 0) and (*h* −2.5 0) reflections are given in panels (b) and (c), respectively, which are the selected regions shown in panel (a).

Focusing on detailed results of structural refinements, Figure 2b shows the quality‐of‐fit parameters, R_1_, of the *Pm*, *P*2_1_/*m*, and $Im\bar{3}$ space groups as a function of temperature. In monoclinic cells with beta angles near 90° there is the possibility of pseudomerohedral twining due to rotation of the unit cell by 180° about the *a* or *c* axes. Models including this type of twinning were examined. For *Pm* and *P*2_1_/*m* space groups, the solid squares indicate the R1 fitting parameters which incorporate a pseudomerohedry^[^ ^28^ ^]^ twin law representing a twofold rotation about the monoclinic *a*‐axis, while the open squares are the R1 parameters of racemic twinning for *Pm* structure and no twinning for *P*2_1_/*m* structure. The racemic twinning in the *Pm* structure indicates the existence of a temperature‐independent 50/50 distribution of inverted and non‐inverted polar domains by volume in the studied temperature range, as expected for an unpolarized sample.^[^ ^29^, ^30^ ^]^ The temperature‐dependent pseudomerohedry twin domain fraction is given in Figure 2c. Below 410 K, possible space groups are the non‐centrosymmetric *Pm* or the centrosymmetric *P*2_1_/*m* space groups. Intensity statistics strongly favored the non‐centrosymmetric space group. However, incorporation into the refinement of the pseudomerohedry twin law and a racemic twin fraction significantly improves the R1 parameters between 260 and 410 K for both models leading to similar R1 values. Even with the lower number of parameters utilized in the *P*2_1_/*m* model (111 parameters for *P*2_1_/*m* model and 209 parameters for *Pm* model), refinement of the *P*2_1_/*m* model converged to better residual values, 4.4/−4.8 e Å^−3^, than that of *Pm* structure, 4.8/−5.3 e Å^−3^, at 350 K. In addition, a careful survey of the data sets revealed systematic extinctions of (0*k*0) reflections for *k* = 2*n* + 1 above 300 K. The centrosymmetric monoclinic space group *P*2_1_/*m* is thus uniquely defined as the structure between 300 and 410 K. The pseudomerohedry twin domains are formed as a result of the *Pm* to *P*2_1_/*m* phase transition. This continuous transition onsets at ≈260 K and is completed at ≈300 K. The inset in Figure 2c reveals an abrupt change of twin domain fraction at ≈350 K indicating a first‐order phase transition. A comparison of *P*2_1_/*m* structure and the early reported *P*4/*mbm* structure solved from the same data is given in Table S2B, Supporting Information. The maximum displacement is 0.18 Å for Cs and 0.32 Å for Br. Figure S6, Supporting Information, shows a good match of the experimental reciprocal space images with the simulated pattern of the *P*2_1_/*m* structure solution.

In comparison to powder diffraction measurements, the single‐crystal diffraction method is better suited to distinguish between the *P*4/*mbm* and *P*2_1_/*m* space groups or between the $Pm\bar{3}m$ and $Im\bar{3}$ space groups as shown in Figures S7 and S8, Supporting Information. In powder diffraction data (see Figure S12A, Supporting Information), weak superlattice peaks overlap with peaks derived from the smaller approximate unit cells especially at a high 2*θ* angle. As shown in Figure S9, Supporting Information, only strong reflections can be captured and utilized with previously proposed unit cell dimensions. While in this work, we utilized all statistically significant reflections for structural refinements. The unfitted reflections in the early work have half‐integer (*hkl*) values based on the old unit cell dimensions. These half‐integer reflections are about 10^2^ to 10^4^ times weaker in intensity than the integer ones and can be indexed only with a larger unit cell. Capturing the half‐integer reflections requires synchrotron‐based XRD measurements and high‐quality single‐crystal samples. Utilizing the weak reflections exclusively in structural refinement yields the same atomic positions that are refined with the dominant reflections at high temperature as shown in Table S3, Supporting Information. The *F*_obs_ versus *F*_calc_ plots for the superlattice peaks and main peaks in *Im*‐3 structure are given in Figure S8f, Supporting Information. Thus, the half‐integer peaks indeed come from the same structure as the main peaks. Figures S8g and S7h, Supporting Information, show a good match of the experimental reciprocal space images with the simulated pattern of the $Im\bar{3}$ structure solution. Accounting for these weak reflections will lower the symmetry by including additional distortions to the previously assumed higher‐symmetry structures. The atomic displacement of the newly proposed structures in this work compared to the structures solved from the same data but in early reported space groups are given in Table S2, Supporting Information. At 450 K, the $Im\bar{3}$ space group incorporates distortions of 0.11 Å in the Br position away from the high‐symmetry special positions in the $Pm\bar{3}m$ structure.

For temperatures below 300 K, as shown in Figure S10, Supporting Information, the reciprocal lattice images reveal half‐integer reflections as seen in early work.^[^ ^12^, ^13^, ^14^, ^15^ ^]^ These weak reflections are also 10^2^ to 10^4^ times weaker than the main peaks at high temperatures. Utilization of a subset of the data including only high‐intensity reflections will yield the *Pnma* space group at room temperature as found in previously published works. Significant systematic absence violations are observed in all of the possible orthorhombic space group solutions with this unit cell dimension, $\sqrt{2}a_{p}\times \sqrt{2}a_{p}\times 2a_{p}$. The violations are evident as peaks with intensities that are about 10^2^ lower than the dominant peak intensities. In particular, the currently accepted space group, *Pnma*, has 378 systematic absence violations at 300 K. The list of systematic absence violation are given in Table S4 and Figure S11, Supporting Information. On the other hand, careful considerations showed that all these previously unfitted half‐integer reflections in the orthorhombic model can indeed be indexed on a primitive monoclinic supercell with cell dimension ≈2*a*_p_ × 2*a*_p_ × 2*a*_p_. These weak reflections as well as the systematic absence violations are the signature of the weak distortions breaking the center of symmetry in *Pnma* structure model. As shown in Figure S12, Supporting Information, the simulated powder patterns of the low‐temperature models indicate that these weak reflections are rather critical to distinguish the appropriate space group of the low‐temperature phase. These points to the need for single‐crystal measurements for complete structural solutions. The structure which accounts for all reflections in the diffraction data for temperatures below 300 K is *Pm* space group. Figure  S13B, Supporting Information, shows a good match of the experimental reciprocal space images with the simulated pattern of the *Pm* crystal structure solution. Compared to the *Pnma* structure, the main distortion in *Pm* structure occurs in Cs and Br atoms as shown in Table S2C, Supporting Information. Representative structural parameters from the single crystal structure solutions are given in Tables S5– S10, Supporting Information. Supporting the low‐temperature assignments, rotational anisotropy second harmonic generation experiments indicate that the true space group has no inversion center for the low‐temperatures phase between 290 and 190 K.

Rotational anisotropy second harmonic generation (RA‐SHG) measurements were carried out to detect the crystal structure characteristics of low‐temperature phase below 300 K. It is noted that in our Raman, single‐crystal diffraction, and pair distribution function measurements, a centrosymmetric‐to‐noncentrosymmetric transition occurs at ≈300 K. Consequently, our RA‐SHG measurements in this work were conducted at and below 290 K in a cryostat to ensure that the sample was in the low‐temperature phase. This contrasts with previous SHG measurements conducted at room temperature with no temperature control.^[^ ^31^ ^]^ In another recent SHG experiment, the SHG photon energy was well below the band gap of CsPbBr_3_, resulting in very low SHG conversion efficiency.^[^ ^32^ ^]^ For this work, the geometry of the RA‐SHG experiments is shown in **Figure** **4**a where oblique incidence with a small incident angle *θ* was used. Incident and reflected light can be selected to be either parallel (*P*) or perpendicular (*S*) to the scattering plane. The 800 nm laser beam was focused on the *ab* plane of the CsPbBr_3_ sample, with the full width at half maximum (FWHM) of the focused beam being ≈20 µm and the penetration depth of 0.42 µm at 800 nm (0.12 µm at 400 nm).^[^ ^33^ ^]^ The spectrum of the incident fundamental light shown in Figure 4b exhibits a central wavelength at 800 nm with a FWHM of 41 nm, which corresponds to the SHG spectrum centered at 400 nm with a FWHM of 15 nm. This fact together with a 400 nm center, 40 nm FWHM bandpass filter before the detector ensures that any detected signal belongs to the SHG instead of the two‐photon fluorescence around 520 nm^[^ ^33^ ^]^ from the sample. The instrument utilized in the experiments is sensitive to SHG signals at levels as small as 10 fW.

**Figure 4:**
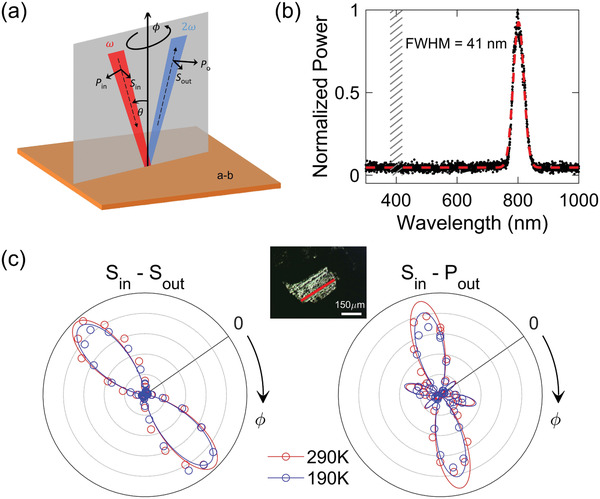
a) Diagram of the RA‐SHG setup. The fundamental beam is focused on the *ab* plane of the crystal with a fixed small incident angle *θ*. The scattering plane rotates about the surface normal by an angle *ϕ*. b) The spectrum of the incident fundamental light, centered at 800 nm with a FWHM of 41 nm. The shaded area at 400 ± 20 nm represents the bandpass region of the filter used to only collect SHG signals. c) The RA‐SHG patterns in *S*_in_ − *S*_out_ and *S*_in_‐*P*_out_ channels under 190 K (blue) and 290 K (red). *ϕ* = 0 represents the direction of a natural edge of the sample. SHG barely shows any difference between data sets at these two temperatures. Inset: the picture of a measured CsPbBr_3_ sample. The red bar marks its natural edge.

The measured RA‐SHG signals are given in Figure 4c. It is observed that the signals in *S*_in_ − *S*_out_ and *S*_in_ − *P*_out_ channels taken at 290 and 190 K have almost identical patterns with slightly different intensity levels. Such features are seen across the whole temperature range from 290 to 190 K, indicating that there is only one structural phase in this temperature region. Moreover, by calculating the field strengths of the incident fundamental and reflected SHG signal, we found that the average SHG susceptibility tensor magnitude is about 0.1 pm V^−1^ which is in the range of typical electric dipole SHG susceptibility values^[^ ^34^ ^]^ and is about three orders of magnitude larger than those of electric quadrupole.^[^ ^35^ ^]^ Note that the presence of multiple domains in the measurement volume will spread the radiation emission of the SHG signal over a broader angular range compared to that of the corresponding single domain crystal.^[^ ^36^ ^]^ Hence, the existence of strong SHG spectra (Figure 4c, *S*_in_ − *S*_out_ and *S*_in_ − *P*_out_ channels) from the electric dipole process demonstrate that the crystal possesses a non‐centrosymmetric structure over the temperature range examined from 290 to 190 K.

To understand the local structure, PDF and X‐ray absorption measurements were also conducted. X‐ray PDF measurements were conducted between 10 and 500 K. Fits to the PDF data were conducted over the real space range 2 ⩽ *r* ⩽ 30 Å compared to the single unit cell averaged parameters explored in the single‐crystal diffraction study above. The PDF goodness‐of‐fit parameter *Rw* was obtained as a function of temperature for a range of space groups explored in the single‐crystal methods. (The *Rw*=$\left\{\frac{\Sigma_{i=1}^{N}{\left[G_{\text{Obs}}\left(r_{i}\right)-G_{\text{Calc}}\left(r_{i}\right)\right]}^{2}}{\Sigma_{i=1}^{N}\omega \left(r_{i}\right){\left[G_{\text{Obs}}\left(r_{i}\right)\right]}^{2}}\right\}$ for PDF data was scaled by the number of independent parameters minus the number of free fitting parameters.^[^ ^37^ ^]^) In **Figure** **5**a, we show the temperature‐dependent *Rw* parameters for orthorhombic models (*P*2_1_2_1_2_1_, *Pna*2_1_, and *Pnma*) compared to the high‐symmetry tetragonal (*P*4/*mbm* and *I*4/*m*) and cubic ($Im\bar{3}$ and $Pm\bar{3}m$) models. In this PDF analysis, the orthorhombic space groups serve as simple models of the low‐temperature structure to assess temperature‐dependent trends in structure.

**Figure 5:**
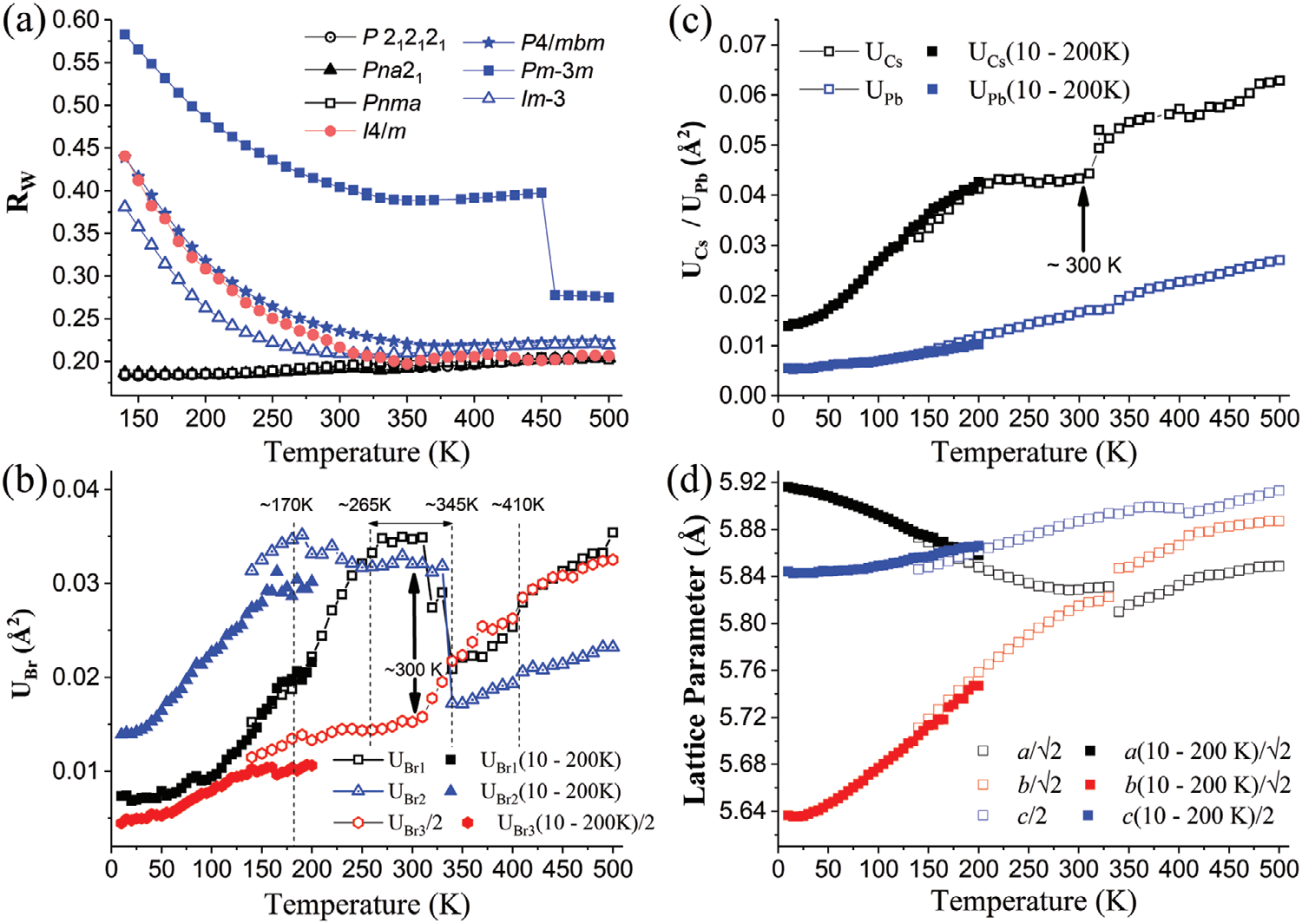
Results for local pair distribution function measurements. a) The goodness‐of‐fit parameter, *Rw*, versus temperature for different models, *P*2_1_2_1_2_1_, *Pna*2_1_, *Pnma*, *P*4/*mbm*, $Pm\bar{3}m$, *I*4/*m*, and $Im\bar{3}$. Atomic displacement parameters as a function of temperature for the Br, Cs, and Pb sites derived from the *Pna*2_1_ model are shown in (b) and (c). In panels (b) and (c), solid symbols and open symbols are given for two independent data sets collected. The ADP parameters reveal clear structural changes at ≈170 K and ≈410 K. Broad regions of structural change are seen in ADPs between 265 and 345 K for the Br sites and between 170 and 300 K for the Cs sites (in panes (b) and (c)). d) PDF derived temperature‐dependent lattice parameters between 10 and 500 K with an abrupt change near 350 K.

Examining Figure 5, it is seen that the $Pm\bar{3}m$ cubic structure does not represent the local structure for the entire temperature range measured. With reduced temperature, the best model is the *I*4/*m* tetragonal structure between 350 and 500 K followed by the *Pna*2_1_ or *P*2_1_2_1_2_1_ space groups for temperatures below ≈350 K. Expanding the temperature range down to 10 K reveals multiple transitions if the Br and Cs atomic displacement parameters (ADPs) are examined. In Figure 5b, we show the Br and Cs ADPs, U_Br_ and U_Cs_. Examination of the Br ADPs (for Br1, Br2, and Br3 sites) reveals a continuous transition near 170 K, a transition which onset near 300 K leading to an abrupt change near 350 K and a kink near 410 K. The transitions near 300 and 170 K are particularly clear in the Cs and Pb ADPs shown in Figure 5c. The 170 K transition is seen in the Raman data (see Figure 1c) as well as in these PDF results. No space group change is indicated for the transitions near 170 K. Hence, this is consistent with an order–disorder type continuous transition. We also note that in the single‐crystal diffraction data discussed above, no new peaks are seen when comparing reciprocal space images at 200 and 120 K. Figure 5d gives the full PDF derived temperature‐dependent lattice parameters between 10 and 500 K. Panels in Figure 5b,c reveal broad regions of structural change seen in ADPs between 265 and 345 K for the Br sites and between 170 and 300 K for the Cs sites. The lower temperature limit in the Cs case is due to its higher mobility in the lattice compared to Br. This observation is consistent with the observed formation of twinning domains in the diffraction measurement shown in Figure 2c. In contrast to the continuous changes observed in bulk lattice parameters derived from single‐crystal diffraction measurement for all phase transition, the Cs, Pb, and Br ADPs, and PDF derived *a* and *b* lattice parameters change abruptly at ≈350 K. In the bulk structural measurement, the atomic pairs are averaged over the whole volume of the measured single crystal. Since the wavelength (*λ* = 0.41328 Å) used in our single‐crystal diffraction measurements gives an attenuation length of ≈110 µm which is significantly larger than the crystal dimensions. While local structural probes can resolve the local distortions into distinct atomic pairs. This observation in local structure indicates an abrupt change of domain structure revealing the first‐order nature of the phase transition at ≈350 K. It also supports the existence of local polar fluctuations reported previously.^[^ ^23^ ^]^ We suggest here that such fluctuations may drive the isostructural first‐order transition at 350 K. Significant deviation in *Rw* between the tetragonal and orthorhombic models occurs below 410 K indicating the existence of a local monoclinic phase for temperatures up to 410 K consistent with the single‐crystal diffraction measurement results. These results are compatible with the recent nano‐scale X‐ray diffraction imaging results which found that the domains formed at 350 K are partially stable subject to cycling to lower and higher temperatures.^[^ ^38^ ^]^

To explore specific atomic correlations in this material, X‐ray absorption measurements were conducted between 20 K and room temperature. Three‐component fits (Br–Pb, Br–Cs, and Br–Br pairs), over the *R*‐space range 2.0 ⩽ *r* ⩽ 4.0 Å, were made at each temperature between 20 and 95 K, and a single‐component fit (Br–Pb) was made for data above 95 K. The shells beyond the nearest neighbor Br–Pb shell (Br–Cs and Br–Br) are found to be suppressed for higher temperatures (above ≈100 K, see Figure S16, Supporting Information). The extracted widths for the Br–Pb shell were then fit to a simple Einstein model: $\sigma^{2}\left(T\right)=\sigma_{0}^{2}+\frac{\hbar^{2}}{2\mu K_{E}\theta_{E}}\coth\left(\frac{\theta_{E}}{2T}\right)$,^[^ ^39^ ^]^ where *μ* is the reduced mass for the atomic pair, and $\sigma_{0}^{2}$ represents the static disorder. This simple model represents the atomic pair motion as harmonic oscillations of a single effective frequency proportional to *θ*_*E*_. The fits yield an Einstein temperature of 104 K corresponding to an effective oscillator frequency of 72 cm^−1^, indicating the extreme softness of the material. The low‐temperature *σ* ^2^ values indicate static disorder for Br–Cs pairs, which is more than 20 times that for the first neighbor Br–Pb pairs and more than two times that for Br–Br pairs (Figure S16, Supporting Information). Examination of the Br–Cs pair distribution derived from the single‐crystal data at low temperature, reveals a broad spread in Br–Cs atomic distances consistent with this result (Figure S17, Supporting Information). There is a large spread in the Br–Cs distribution in the *P*2_1_2_1_2_1_ space group. This spread in positions becomes less broad at low temperatures (see Figure S18, Supporting Information). Our molecular dynamics simulations reveal a larger dynamic change in Br–Pb and Br–Cs pair distributions in going from 100 to 250 K, consistent with the proposed order–disorder transition (Figure S18, Supporting Information). The Br–Cs correlations derived from these simulations show the most significant change with temperature consistent with the large thermal component to the disorder.

To understand the stable structural phases under strain, which may be present when films are grown on substrates with lattice mismatch, we conducted pressure‐dependent structural measurements. **Figure** **6** exhibits the complementary high‐pressure Raman and XRD measurements conducted between ambient pressure and 17 GPa. Figure 6b shows the pressure dependence of specific Raman peaks revealing the onset and disappearance of features at the phase transitions. The sharpening of the peak near 70 cm^−1^ in low‐temperature ambient pressure measurements coincides with the appearance of the additional peak near 70 cm^−1^ above 1 GPa. The very low pressure of the transition indicates the softness of the material. In previous pressure‐dependent Raman measurements, the transition at ≈1 GPa has been associated with Pb–Br distance length shrinkage and distortions of the PbBr_6_ polyhedra.^[^ ^40^ ^]^ Recall that the Raman peaks at 70 cm^−1^ correspond to Cs atom shear‐type motion triggered by asymmetric distortion modes of the PbBr_6_ polyhedra. Hence, comparison of temperature‐dependent and pressure‐dependent Raman data suggests very low pressure recovers the low‐temperature behavior of this system possibly with suppression of the disorder which onsets for temperatures above 170 K. It is expected that moderately straining these materials may lead to more stable phases with lower levels of electron scattering with better transport properties. High‐pressure diffraction results are given in Figure 6c,d. In Figure 6d, an abrupt (first‐order) transition is found near 1 GPa which is followed by a continuous transition that is completed near 2 GPa. Continuous transitions are also observed near 6 and 13 GPa. Over the full range studied, we found five distinct structural phases (phase I to V). Note that the transition near 1 GPa results in a splitting of peaks consistent with symmetry reduction below the ambient pressure space group.

**Figure 6:**
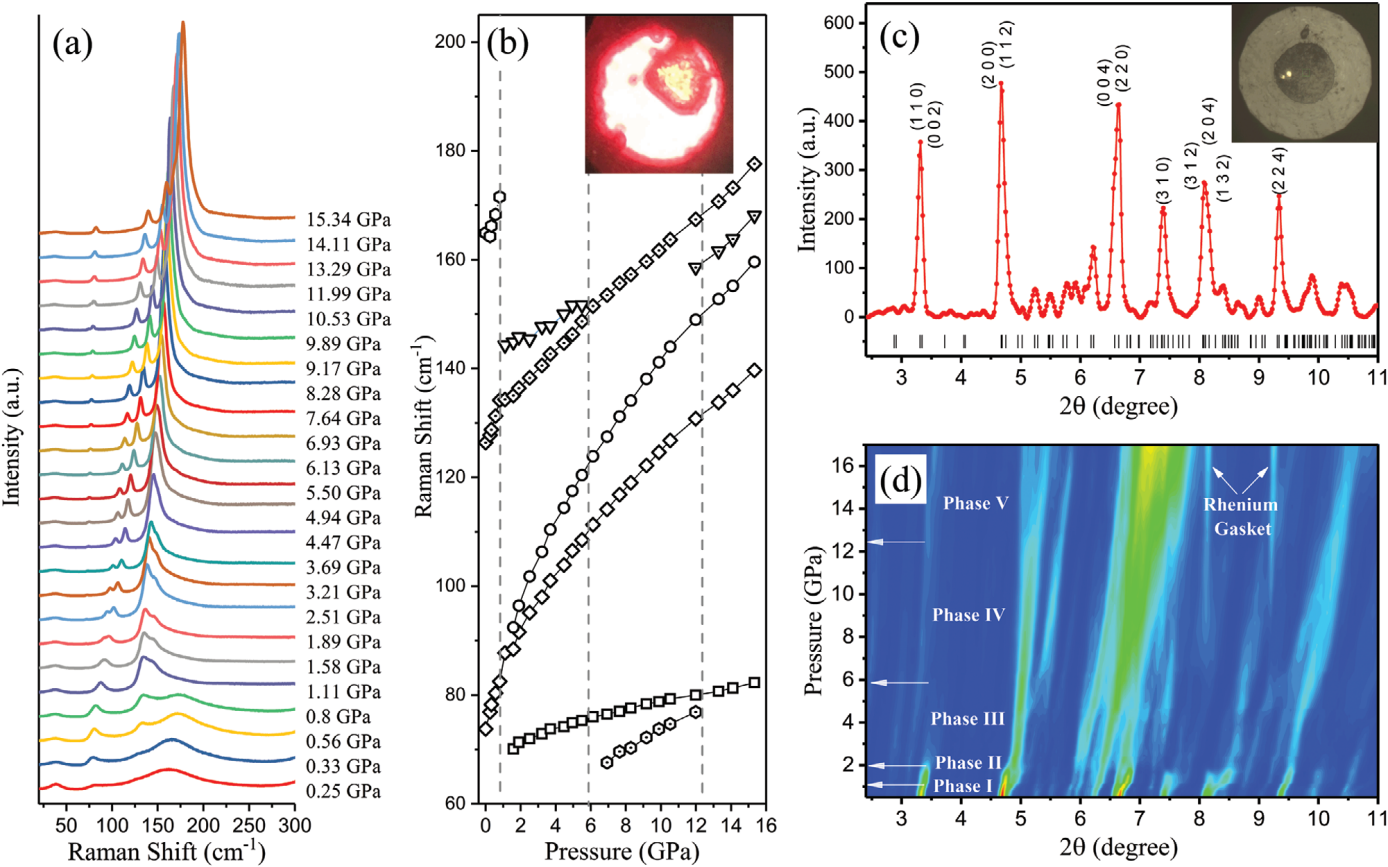
High‐pressure structural changes at room temperature. a) High‐pressure Raman spectra for pressures between 0.6 and 15 GPa. The fitted Raman peak positions are shown in (b), indicating three phase transitions. The inset in (b) shows the sample in the diamond anvil cell with spot from a 646 nm laser. c) Representative high‐pressure powder X‐ray diffraction pattern measured at 0.6 GPa. The inset shows the sample in the diamond anvil cell. d) The 2D intensity plot of the pressure‐dependent X‐ray diffraction patterns indicates transitions at ≈1, 2, 6, and 13 GPa. The pressure‐dependent structural phases are labeled phase I to phase V.

To examine the behavior at low pressures, we expand the diffraction map between 0.6 and 3 GPa in **Figure** **7**a and note that three phases are present. The region between 1 and 2 GPa reveals strong splitting of diffraction and Raman peaks. In the top panel of Figure 7c, placing the sample on a glass slide yields Raman spectra characteristic of the normal bulk phase. Manually compressing the sample between a pair of glass slides and then remeasuring the spectra after removing the top slide produces a phase different from the original ambient pressure phase as shown in the lower panel. These additional features in the spectra are indicated as * symbols. By linearly extrapolating the high‐pressure phase II and phase III frequencies back to the ambient pressure in Figure 7b, we see that the new features are from a stabilized component of phase II in the sample. Hence phase II can exist at ambient pressure as a metastable phase after pressure release. This metastable form of phase II is easy to achieve by releasing pressure rapidly after compression with glass slides. Measurements conducted 2 weeks after slide compression of the samples reveal the same spectra. On the other hand, after hydrostatic compression up to 17 GPa, slow release of pressure produces the original ambient pressure Raman and XRD patterns. Very low pressures can stabilize new phases and some of which will be stable under ambient conditions. These phases can be accessed when the material is prepared as a thin film since substrate‐induced strain can readily place samples under these low pressures. We note that all observed transitions are reversible. Also, no amorphous phase is observed in the pressure range explored unlike the results on commercial polycrystalline materials examined in previous work.^[^ ^40^ ^]^

**Figure 7:**
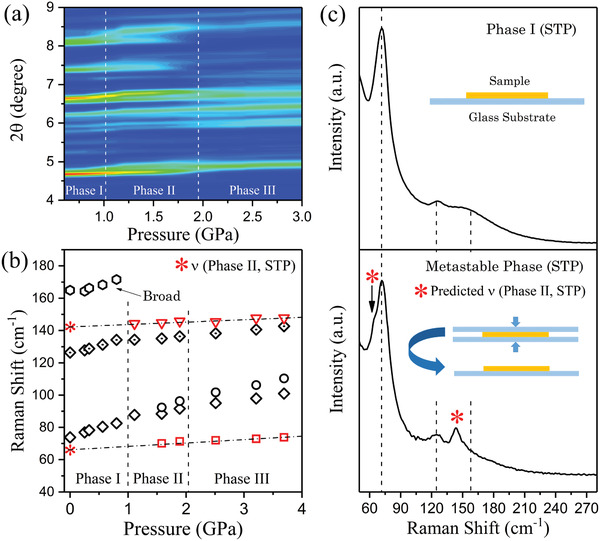
a) Expanded region of the 2D X‐ray diffraction map between 0.6 and 3 GPa showing a new phase that covers the region between 1 and 2 GPa. b) The pressure‐dependent Raman peak positions with dot‐dashed lines giving predicted positions at standard temperature and pressure (STP) from linear extrapolation of the phase II peaks, indicated with * symbols. In the top panel of (c), the Raman spectrum of the original sample is shown while the spectrum of a sample after compression between glass slides is given in the bottom panel. The appearance of additional peaks is indicated by the * symbols. Compression between the glass slides brings the samples into phase II and a part of the sample is maintained in this phase after release of pressure (metastable phase II).

The combined results of our experiments point to four distinct structural phases with cell dimensions ≈2*a*_p_ × 2*a*_p_ × 2*a*_p_ as temperature changes between 500 and 10 K: a high‐temperature cubic phase (above ≈410 K), a high‐temperature monoclinic phase (between ≈410 K and ≈350 K), a low‐temperature monoclinic phase (between ≈350 and ≈300 K), and finally, a low‐temperature monoclinic phase (below ≈300 K). An isostructural phase transition is observed at ≈350 K. An order–disorder transition is revealed at ≈170 K by the loss of higher‐order pair correlations in the X‐ray absorption fine structure (XAFS) data and changes in the PDF ADPs. For all phase transitions, changes are evident in at least two of the independent measurements conducted with the ≈170 K transition exclusively apparent in local structural studies. The phase transition near 300 K is found to be second‐order and the other two higher‐temperature phase transitions are found to be first‐order in nature. The lower temperature transitions have not been identified in previous structural studies. The proximity of the second‐order transition to room temperature suggests that room temperature measurements without temperature control may lead to uncertainty in the space group assignment (centrosymmetric vs non‐centrosymmetric). The local structural measurements indicate that the presence of distortions supports locally non‐centrosymmetric symmetry for temperatures up to at least 500 K. The local structure is never in a cubic phase between 10 and 500 K. In terms of the bulk structure, the phase below 300 K is non‐centrosymmetric. The observed systematic absence violations and superlattice reflections reveal the inadequacy of the currently accepted room‐temperature space group *Pnma*. We note that recent work on the CsPbI_3_ system assumes the approximate space group assignments.^[^ ^41^ ^]^ High dynamic‐range synchrotron‐based data sets should be collected to understand this general class of materials. Detailed studies such as those conducted here are needed to determine the correct space groups and corresponding physical properties. Such measurements will assist in the development of more accurate theoretical models of these important materials.

3

Understanding the basic physics underlying the properties of this material requires an accurate determination of the crystal structure. This is required to develop accurate potentials to predict the finite temperature properties such as transport and optical absorption. These critical properties are very sensitive to subtle structural details. New space group assignments are made for the temperature range from 100 to 500 K. Structural measurements combined with second harmonic generation measurements reveal the absence of inversion symmetry below 300 K. In this temperature region, the true unit cell is a monoclinic *Pm* cell. The space group is *P*2_1_/*m* between ≈300 K and ≈410 K and $Im\bar{3}$ above ≈410 K. The structural parameters provided in this work will assist in the development of accurate models leading to the prediction of new and more efficient analogs of the all‐inorganic CsPbBr_3_ system. The first‐order nature of the ≈410 K and ≈350 K transitions will impact transport properties if these materials are cooled rapidly once heated. Rapid cooling from above 350 K may freeze in the high‐temperature phases. High‐pressure experiments indicate multiple phase transitions at low pressure (near 1 and 2 GPa). These new phases will influence the properties of films grown on substrates with significantly different lattice constants from that of ambient CsPbBr_3_. At low pressure, a phase is observed which can exist as a metastable phase at ambient pressure.

## Experimental Section

### Differential Scanning Calorimetry

Differential scanning calorimetry measurements were conducted under flowing N_2_ gas using a Perkin Elmer DSC 6000. Measurements were made with a cooling/heating rate of 2 K min^−1^.

### Raman Scattering Measurements

Ambient pressure temperature‐dependent Raman spectra were measured with an excitation wavelength of 780 nm in backscattering geometry using a Thermo Scientific DXR Raman Microscope. A 50× objective was used with a 15 mW laser power setting. The sample was found to be stable under this laser power after tests were done on a range of laser power values (0.1–15 mW). These measurements were conducted at the NJIT York Center. Samples return to the original phase after heating up to the maximum temperature of 830 K used in the experiments. High‐pressure Raman measurements were conducted at the National Synchrotron Light Source II (NSLS‐II) beamline 22‐IR‐1. Measurements were conducted in a symmetric cylindrical diamond cell with (100) oriented diamonds. For all Raman measurements, no change in the spectra was observed over time at a given pressure. Each pressure data set is comprised of sixty 10‐s scans.

### X‐Ray Single‐Crystal Diffraction Measurements

Diffraction measurements were conducted on an as‐grown cube‐shaped single crystal of edge ≈50 µm at the advanced photon source (APS) beamline 15‐ID‐D at Argonne National Laboratory using a wavelength of 0.41328 Å (30 keV). The data were collected with a PILATUS 1M detector between 100 and 450 K in steps of 10 K (data are for increasing temperature). The NSF ChemMatCARS beamline is an undulator beamline. An undulator source does not output a continuous X‐ray spectrum but a sharply peaked spectrum centered at the set energy, which was 30 keV in this case. In addition, the beamline utilized a Si (111) double crystal monochromator. The Si (222) Bragg reflection was forbidden. More importantly, the beamline had a harmonic rejection mirror to suppress the photons with energies above 30 keV. Hence the combination of tuned undulator energy, the use of a Si (111) monochromator, and a harmonic rejection mirror made Bragg peaks due to the *λ*/2 (60 keV) contamination impossible.

### Second Harmonic Generation Measurements

The reflected SHG intensity was recorded as a function of the azimuthal angle *ϕ* (Figure 4). The incident ultrafast light source was of 50 fs pulse duration and 200 kHz repetition rate, and was focused down to a 20 µm diameter spot on the sample with a fluence of ≈1 mJ cm^−^^2^. The RA‐SHG patterns remain the same on increase of the fluency to ≈2 mJ cm^−^^2^

### Phonon DOS and Molecular Dynamics Simulations

To determine force constants and phonon DOS for CsPbBr_3_, density functional calculations in the projector augmented wave approach were carried out. Full structural optimization was conducted for both lattice parameters and atomic positions. The ground‐state structure was optimized so that forces on each atom were below 2 × 10^−5^ eV Å^−1^. The phonon density of states and phonon displacement modes were derived from the computed force constants. *A*b initio molecular dynamics (MD) simulations were also conducted. A 2 × 2 ×2 orthorhombic supercell (based on the optimized structure obtained above with 160 atoms) was utilized. In the MD simulations, the system temperature was set at 100, 250, and 500 K utilizing the NVT ensemble. MD time steps of 1 fs were used, with ≈2500 time step for each simulation.

### X‐Ray Absorption Fine Structure Measurements

Br K‐edge XAFS spectra were collected at APS beamline 20‐BM at Argonne National Laboratory on single crystals (≈2 mm × 3 mm× 0.5 mm) in fluorescence mode (20 to 125 K). Measurements were done in fluorescence mode on powders at NSLS‐II beamline 7‐BM (120 to 300 K). Data were corrected for self‐absorption. Reduction of the X‐ray absorption fine structure data was performed using standard procedures.

### Pair Distribution Function Measurements

Two independent pair distribution function data sets (140–500 K (run 1) and 10–200 K (run 2)) were collected at NSLS‐II beamline 28‐ID‐2(XPD) at Brookhaven National Laboratory using a wavelength *λ* = 0.1877 Å and *λ* = 0.1872 Å for run1 and run2, respectively. Measurements utilized Perkin Elmer Area detectors with a sample to detector distance of ≈200 mm. Exact detector to sample distances were derived by fits to Ni powder calibration standards. The Ni standard was used to determine set‐up specific parameters (*Q*_damp_ and *Q*_broad_), which were held during data modeling.

### High‐Pressure Powder Diffraction Measurements

High‐pressure powder diffraction measurements were performed at APS beamline 13‐ID‐D (GSECARS) at Argonne National Laboratory. The beam size used was 2.3 µm (vertical) × 3.1 µm (horizontal) with a wavelength of 0.3344 Å. A PILATUS 1M detector was used to collect the diffraction images. The sample‐detector distance was 207.00 mm. The sample‐detector geometry was calibrated with a LaB_6_ powder NIST standard. The measurements were conducted with a diamond cell with 400 µm cutlets. A 200 µm thick rhenium gasket pre‐indented to 42 µm with a 200 µm hole was used as the sample chamber. Neon was used as the pressure transmitting medium. Ruby balls and gold balls were placed near the pressed powder samples. The gold compression curve was used for pressure calibration.

### High‐Pressure Powder Diffraction Measurements

High‐pressure powder diffraction measurements were performed at APS beamline 13‐ID‐D (GSECARS) at Argonne National Laboratory. The beam size used was 2.3 µm (vertical) × 3.1 µm (horizontal) with a wavelength of 0.3344 Å. A PILATUS 1M detector was used to collect the diffraction images. The sample‐detector distance was 207.00 mm. The sample‐detector geometry was calibrated with a LaB_6_ powder NIST standard. The measurements were conducted with a diamond cell with 400 µm cutlets. A 200 µm thick rhenium gasket pre‐indented to 42 µm with a 200 µm hole was used as the sample chamber. Neon was used as the pressure transmitting medium. Ruby balls and gold balls were placed near the pressed powder samples. The gold compression curve was used for pressure calibration.

### Statistical Analysis

All data presented represent the mean values of multiple collected scans. The errors given for measurements were based on standard deviation on these average values. The data given were representative of the crystalline CsPbBr_3_ material used in the measurements. All Raman and high‐pressure diffraction data were shown after background subtraction for better visualization. Specific details for all methods, software, and references used for data processing and analysis are given in the Supporting Information.

## Conflict of Interest

The authors declare no conflict of interest.

## Supporting information

Supporting Information

## Data Availability

Research data are not shared.
