# Supplementary material for: High‐Resolution In‐Situ Synchrotron X‐Ray Studies of Inorganic Perovskite CsPbBr3: New Symmetry Assignments and Structural Phase Transitions
Source: Adv Sci (Weinh). 2021 Jul 11;8(18):2003046. doi: 10.1002/advs.202003046 (PMC8456275; doi:10.1002/advs.202003046)
Supplement: Supplementary file 1 — Supporting Information [file ADVS-8-2003046-s001.pdf]

© 2021 Wiley-VCH GmbH

ADVANCED SCIENCE
Open Access

Supporting Information

for *Adv. Sci.,* DOI: 10.1002/advs.202003046

High-Resolution In-situ Synchrotron X-ray Studies of Inorganic Perovskite CsPbBr3: New Symmetry Assignments and Structural Phase Transitions

*S. Liu, A. R. DeFilippo, M. Balasubramanian, Z. Liu, S. G. Wang, Y-S. Chen, S. Chariton, V. Prakapenka, X. Luo, L. Zhao, J. San Martin, Y. Lin, Y. Yan, Y. S. Ghose, and T. A. Tyson**

High-Resolution In-situ Synchrotron X-ray Studies of Inorganic Perovskite $CsPbBr_3$: New Symmetry Assignments and Structural Phase Transitions

S. Liu[1], A. R. DeFilippo[1], M. Balasubramanian[2], Z. Liu[3], S. G. Wang[4], Y-S. Chen[4], S. Chariton[4], V. Prakapenka[4], X. Luo[5], L. Zhao[5], J. San Martin[6], Y. Lin[6], Y. Yan[6], Y. S. Ghose[7], and T. A. Tyson[1]

[1]Department of Physics, New Jersey Institute of Technology, Newark, NJ 07102, USA
[2]Advanced Photon Source, Argonne National Laboratory, Argonne, IL 60439, USA
[3]Department of Physics, University of Illinois at Chicago, Illinois 60607-7059, USA
[4]Center for Advanced Radiation Sources, University of Chicago, Argonne, IL 60439, USA
[5]Department of Physics, University of Michigan, Ann Arbor, MI 48109, USA
[6]Department of Chemistry and Biochemistry, San Diego State University, San Diego, CA, 92182, USA
[7]National Synchrotron Light Source II, Brookhaven National Laboratory, Upton, NY 11973, USA

(Supplementary Document)

$PbBr_2$ (1.83 g, 5 mmol) and CH_3NH_3Br (560 mg, 5 mmol) were dissolved in 50 ml of dimethylformamide. The solution was heated slightly to obtain a transparent solution. This solution was further filtered through a compacted Celite column and the filtrate was collected. Two milliliters of this solution was transferred into an inner vial (5 ml in total vial volume) that was placed in a larger outer vial (25 ml in total volume) with 5 ml of toluene inside. Finally, the outer vial was carefully sealed. The diffusion of toluene from the outer vial into the inner vial was slow, and the crystallization process was maintained in a dark and undisturbed environment for at least three days. Orange block-shaped single crystals were obtained and characterized by X-ray diffraction. For powder sample derived experiments, crystals samples were crushed and sieved to obtain ~ 400 mesh powders. All measurements are based on crystal derived materials.

Differential scanning calorimetry measurements were conducted under flowing N_2 gas using a Perkin Elmer DSC 6000. Measurements were made at a cooling/heating rate of 2 K/min.

The rotational anisotropy second harmonic generation (RA-SHG) measurements were performed with the geometry shown in Fig.4(a) and spectrum of the incident beam shown in Fig. 4(b). The reflected SHG intensity was recorded as a function of the azimuthal angle ϕ, while selecting either S_{in}-S_{out} or S_{in}-P_{out} polarization channels. In this experiment, the incident ultrafast light source was of 50 fs pulse duration and 200 kHz repetition rate, and was focused down to a 20 μm diameter spot on the sample and at a power of 0.7 mW, corresponding to a fluence of ~ 1 mJ/cm^2. The intensity of the reflected SHG was measured with a single photon counting detector.

Severe laser-induced lattice dynamics is not observed in our measurements on $CsPbBr_3$. RA-SHG patterns from the same sample at 290 K and the S_{in}-S_{out} channel, taken under different optical fluence 1.1 and 2.3 mJ/cm^2, remain unchanged within the uncertainty level of the measurements. This indicates minimal lattice perturbation from the laser. Furthermore, the pulsed fs-laser source used in the RA-SHG experiments is of a 200 kHz repetition rate, corresponding to 5 μs separations between adjacent pulses. It is unlikely that the photoexcited lattice change/dynamics, if any, have a recovery timescale of microseconds making it detected by subsequent pulses.

Ambient pressure temperature-dependent Raman Spectra were measured with an excitation wavelength of 780 nm in backscattering geometry using a Thermo Scientific DXR Raman Microscope. A 50× objective was used with the laser power set at 15 mW. The sample was found to be stable under this laser power after tests were done on a range of laser power values (0.1 to 15 mW). Each temperature data set is comprised of one hundred 0.2-second scans. A Linkam Scientific THMS600 stage was used to measure the temperature-dependent Raman spectra. Only warming data are shown. Samples return to the original phase after heating up to the maximum temperature of 830 K used in the experiments. These measurements were conducted at the NJIT York Center.

High-pressure Raman measurements were conducted at the National Synchrotron Light Source II (NSLS II) beamline 22-IR-1 National. Measurements were conducted in a symmetric cylindrical diamond cell with (100) oriented diamonds. The diamond culet size was 500 μm, and tungsten gaskets were used. The pressure medium utilized was methanol:ethanol: water in a 16:3:1 ratio by volume. Pressure calibration was conducted using ruby fluorescence mainline shifts [1]. Pressure calibration measurements were made before and after each Raman spectrum was collected. In addition, calibration measurements as a function of position at multiple points (in the sample region of the gasket) at the highest pressure showed a high level of hydrostatic behavior of the pressure medium. Typical pressure errors are ± 0.10 GPa for pressures below ~ 4 GPa and ~ ±0.20 GPa for pressures above ~4 GPa. For this experiment, the custom micro-Raman system at beamline 22-IR-1 consisted of a 646 nm solid-state laser, a Princeton Instruments liquid-nitrogen cooled PyloN CCD detector, a PI Acton SpectraPro SP-2556 Imaging Spectrograph, and a 20× objective. For all Raman measurements, no change in the spectra was observed over time at a given pressure. Each pressure data set is comprised of sixty 10-second scans.

Diffraction measurements on ~50 μm edge length crystals (cube-like shape) were conducted at the Advanced Photon Source (APS) beamline 15-ID-D (NSF's ChemMatCARS) at Argonne National Laboratory using a wavelength of 0.41328 Å (30 keV). The data were collected with a PILATUS 1M CdTe detector (by DECTRIS, maximum count rate = 10^7 cps/pixel, counter depth =20 bit) between 100 K and

450 K in steps of 10 K (data are for increasing temperature). The data were processed using APEX3 (Bruker, 2016)[2]. The experimental reciprocal space precession images were generated using the same software. The simulated reciprocal space images were obtained using SingleCrystal 4.1.2 (CrystalMaker). The solution and refinement of the data were done using the program Olex2 [3] after the reflections were corrected for absorption using SADABS (with computed attenuation length = 105 μm). Anomalous scattering corrections were induced for all atoms. The values of f′ and f″ values for Br, Cs, and Pb at a wavelength of 0.41328 Å are 0.1889 and 0.9628, -1.7794 and 0.8050, and -1.4094 and 4.2152, respectively. Note that in the case of $CsPbBr_3$, the attenuation length of the x-rays beam with λ = 0.41328 Å (30.000 keV) is ~110 μm. This should be compared with standard Cu Kα (8.046 keV) or Mo Kα (17.48 keV) used in laboratory instruments, which yield attenuation lengths of ~10 μm and ~25 μm, respectively. The single-crystal goodness-of-fit parameters R_1 and wR_2 are defined as $R_1 = \sum||F_0| - |F_c||/\sum|F_0|$ and $wR_2 = \sum w(F_o^2 - F_c^2)^2)/\sum w(F_o^2)^2$, respectively. Detailed representative single-crystal solution results are presented in Tables S3 to S7.

We note that the NSF ChemMatCARS beamline 15-ID-D beamline was operated at 30 keV for these measurements. It is an undulator beamline. An undulator source does not output a continuous x-ray spectrum but a sharply peaked spectrum centered at the set energy, which is 30 keV in this case. In addition, the beamline utilized a Si (111) double crystal monochromator. The Si (222) Bragg reflection is forbidden. More importantly, the beamline has a harmonic rejection mirror to suppress the photons with energies above 30 keV. Hence the combination of tuned undulator energy, the use of a Si (111) monochromator, and a harmonic rejection mirror make Bragg peaks due to the λ/2 (60 keV) contamination impossible.

The final solutions presented are based on a comparison of the experimental reciprocal space images (see Figs. S6, S8, S13B below, for example) with the calculated images in addition to evaluation of the R_1 parameters. All observed Bragg peaks are accounted for in this approach (see Refs in [4] for a systematic approach to single crystal structure solution). Weak low-index reflections are essential to determining space groups [4(b)]. We have also accounted for twinning within the crystal [5].

To determine force constants and phonon DOS for $CsPbBr_3$, density functional calculations in the projector augmented wave approach were carried out utilizing the VASP code [6]. Full structural optimization was conducted for both lattice parameters and atomic positions. The LDA exchange functional (Ceperly and Alder as parameterized by Perdew and Zunger [7]) was used to obtain the relaxed structure. The ground-state structure was optimized so that forces on each atom were below 2 x 10^{-5} eV/Å. The optimized cell was found to be orthorhombic with volume = 8.3876 Å x 11.5197 Å x 7.5612 Å utilized ((4,4,4) gamma centered grid). Calculations for a 2×2×2 supercell with a gamma centered k-space grid were. The force constants were calculated in the frozen phonon approximation. The code Phonopy was utilized to determine the phonon density of states and phonon displacement modes from the force constants (Fig. S3, and Table S1) [8]. Gaussian broadening with full-width at half maximum of 7.1 cm^{-1} was applied to each phonon DOS spectrum shown in Fig. S3(a).

To determine the low-temperature space group by modeling methods (DFT based on VASP), we initiated a structural optimization starting from the 120 K XRD Pm solution ($\sim 2a_P \times 2a_P \times 2a_P$ cell, ((4,4,4) gamma centered k-space grid)). In the first runs, the positions of the Pb atoms were fixed. The positions of all Br and Cs atoms were allowed to move, and the unit cell was free to adjust its shape to reduce the forces on all atoms to be less than 2.5 x 10^{-5} eV/Å. A second optimization cycle was conducted will all atoms and lattice parameters were free to adjust until the forces on atoms were minimized (again to less than 2.5 x 10^{-5} eV/Å). The structural optimization resulted in a monoclinic Pm cell with a = 11.293 Å, b= 11.518 Å, c=11.293 Å and b = 95.91°. We found the energy per CsPbBr3 per formula unit (f.u.) to be lower (E=-18.019 eV/f.u.) for this cell than that derived from fully optimizing a cell ((8,8,4) gamma centered k-space grid)) with dimension ~ $\sqrt{2}\, a_P \times \sqrt{2}\, a_P \times 2a_P$ (E=-17.944 eV/f.u.). These smaller cells result in a Pnma structure after optimization. Note that these calculations generate the zero temperature structure.

Molecular dynamics (MD) simulations were also conducted with the VASP code and projector-augmented wave (PAW) potentials [3]. The simulations were conducted as done in Ref. [9] for $MAPbI_3$ and used a 400 eV energy cutoff. A 2×2×2 orthorhombic supercell (based on the optimized structure

obtained above with 160 atoms) was utilized. For separate MD simulations, the system temperature was set at 100, 250, and 500 K utilizing the NVT ensemble. MD time steps of 1 fs were used, with ~2500 time step for each simulation.

Br K-edge XAFS spectra were collected at APS beamline 20-BM at Argonne National Laboratory on single crystals (~2 mm x 3 mm x 0.5 mm) in fluorescence mode (20 K to 125 K). Higher temperature measurements were done in fluorescence mode with powders at beamline at NSLS-II beamline 7 BM (120 K to 300 K). Data were corrected for self-absorption. Reduction of the x-ray absorption fine-structure (XAFS) data was performed using standard procedures [10]. In the XAFS refinements, to treat the atomic distribution functions on equal footing, the Br K-edge spectra were modeled in R-space by optimizing the integral of the product of the radial distribution functions and theoretical spectra with respect to the measured spectra. Specifically, the experimental spectrum is modeled by, $\chi(k) = \int \chi_{th}(k,r)\, 4\pi r^2 g(r) dr$, where χ_{th} is the theoretical spectrum and g(r) is the real space radial distribution function based on a sum of Gaussian functions ($\chi(k)$ is the measured spectrum) [11] at each temperature (as in Ref. [12]). For each shell fit, the coordination number (N) was held at the crystallographic value, but the position (R) and Gaussian width (σ) was fit to the data. the k-range $1.16 < k < 11.1$ Å^{-1} and the R-range $1.96 < R < 4.00$ Å were utilized. Coordination numbers for the atomic shells were fixed to the crystallographic values. The Gaussian widths and positions were fit for each component

Two independent Pair distribution function (PDF) data sets (140 to 500 K (run 1) and 10 to 200 K (run 2)) were collected at NSLS-II beamline 28-ID-2 (XPD) beamline at Brookhaven National Laboratory using a wavelength λ = 0.1877 Å (run 1) and λ = 0.1872 (run 2). Measurements utilized Perkin Elmer Area detectors with a sample to detector distance of ~200 mm. Exact detector to sample distances were derived by fits to Ni powder calibration standards. The Ni standard was also used to determine set-up specific parameters (Q_{damp} and Q_{broad}), which were held fixed for these samples. The range Q_{mim} = 1.2 Å^{-1} and Q_{max} = 24.5 Å^{-1} (run 1) was used in data reduction. (For run 2 the range was Q_{mim} = 1.2 Å^{-1} and Q_{max} = 22.5 Å^{-1} used.) All samples were measured in 1 mm Kapton capillaries with 50 micron thick walls. Scans

were collected with blank capillaries to determine the background scattering. This background was subtracted from all datasets. The methods utilized for analysis of the PDF data are described in detail in Refs. [13]. For the fits in R-space, the range 2.0 < r < 30 Å was utilized. The time interval between temperature points was ~2 minutes. Combined with the small temperature steps, the approach kept the samples from being in a quenched state. For the PDF curves in Fig. 5(a), $R_W = \left\{\frac{\sum_{i=1}^{N} w(r_i)[G_{Obs}(r_i)-G_{Calc}(r_i)]^2}{\sum_{i=1}^{N} w(r_i)[G_{Obs}(r_i)]^2}\right\}$, where G_{Obs} and G_{Calc} are the observed and calculated PDFs and w is the weighting factor; $w(r_i) = 1/\sigma^2(r_i)$, where σ is the estimated standard deviation on the data-point at position r_i, ref[10(b)]). Note that G(r) is the reduced atomic pair distribution function which oscillates about zero and is obtained directly from the scattering data, $S(Q)$, with $Q = \frac{4\,\pi \sin(\theta)}{\lambda}$. The function $G(r) = \frac{2}{\pi}\int_{Q_{min}}^{Q_{max}} Q(S(Q)-1)\sin(Qr)\,dQ$ is related directly to the standard pair distribution function $g(r)$. $G(r) = 4\pi r \rho_0(g(r)-1)$ where ρ_0 is the number density of atoms. F(Q) in Fig. S14(b) is defined as $F(Q) = Q(S(Q)-1)$. The PDF $G(r)$ includes all of Q-space between the limits of integratonin Q-space and not just at the peak positions. Hence it captures the diffuse scattering [14].

High-pressure powder diffraction measurements were performed at APS beamline 13-ID-D (GESCARS) at Argonne National Laboratory. The beam size used was 2.3 μm (V) x 3.1 μm (H) with a wavelength of 0.3344 Å. A Pilatus 1M CdTe detector (by DECTRIS) was used to collect images. The sample-detector geometry was calibrated with a LaB_6 powder NIST standard. The sample-detector distance was 207.00 mm. The measurements were conducted with a diamond cell with 400 μm culets. A 200 μm thick rhenium gasket pre-indented to ~42 μm (with 200 μm hole) was used as the sample chamber. Neon was used as the pressure transmitting medium and Ruby balls and gold balls were placed near the pressed powder samples. Small pressure steps were enabled with the use of a gas membrane apparatus. At each pressure, 1-second exposures were conducted to acquire images. The sample was measured up to ~18 GPa and then released and remeasured. The ambient pattern was recovered on pressure release. Dioptas [15]

were utilized to integrate the two-dimensional diffraction images (powder rings) to generate the intensity vs 2θ curves.

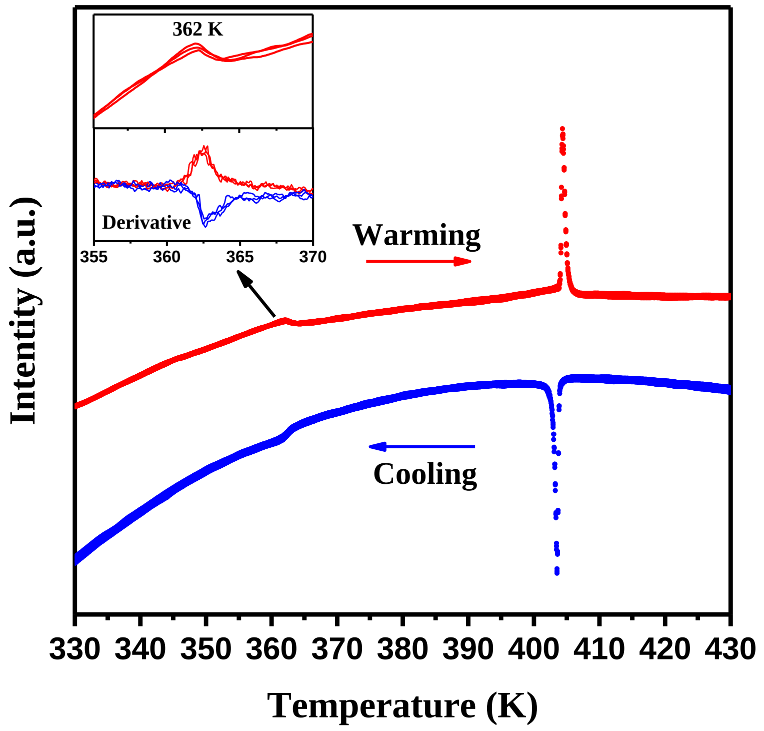

Fig. S1. DSC data showing first-order phase transitions at 362 K and 402 K in $CsPbBr_3$. The inset shows the derivative of the phase transition at 362 K. Both transitions reveal offsets on cooling and warming, pointing to their first-order nature. The inset displays multiple traces of the collected data.

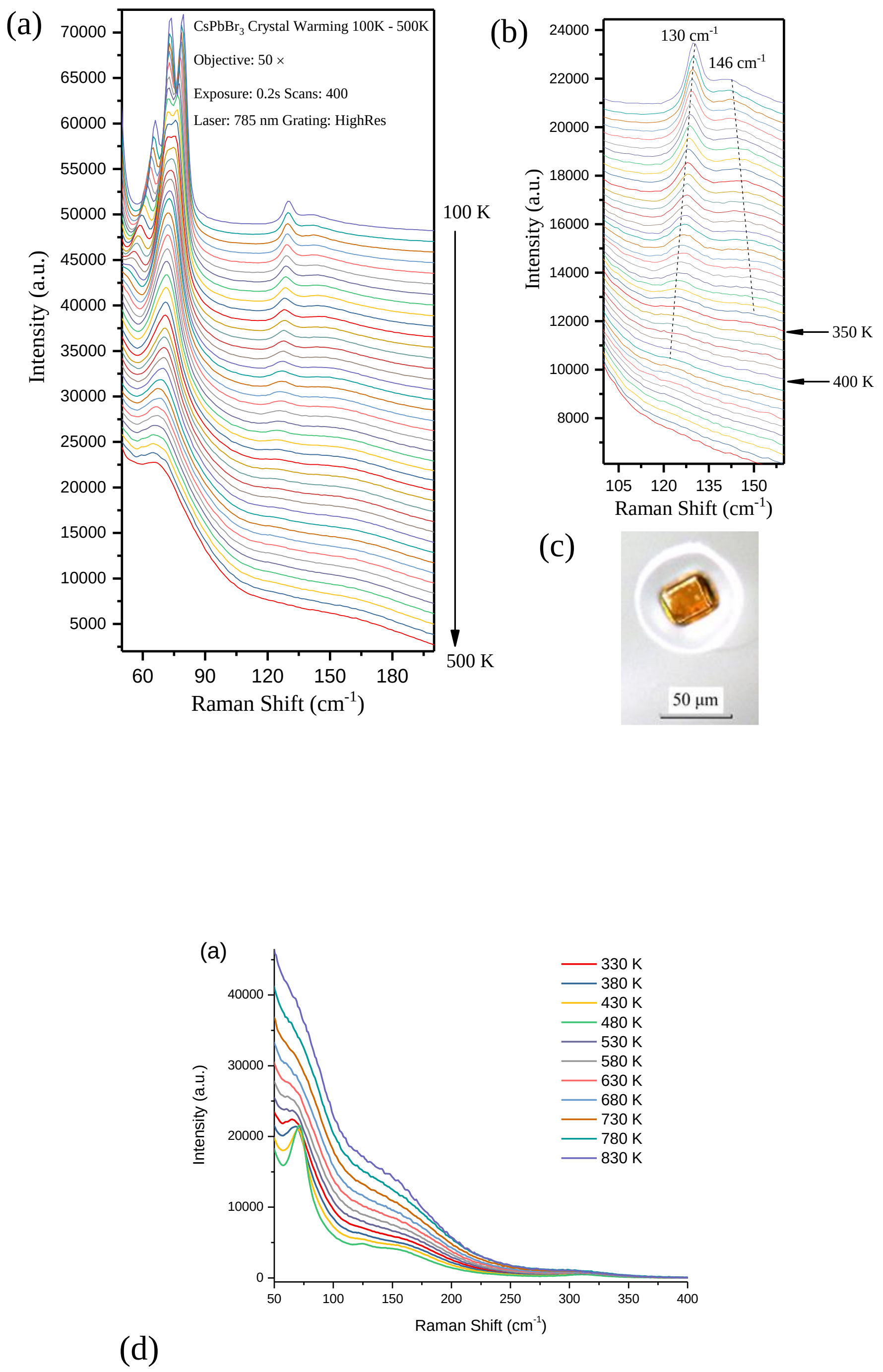

Fig. S2. (a) The Raman spectra of $CsPbBr_3$ from 100 K to 500 K. (b) Expansion near phonon modes at 130 cm^{-1} and 146 cm^{-1}. (c) Representative single-crystal (in oil) used in single-crystal diffraction, Raman, and DSC measurements. (d) High-temperature Raman data between 330 and 830 K.

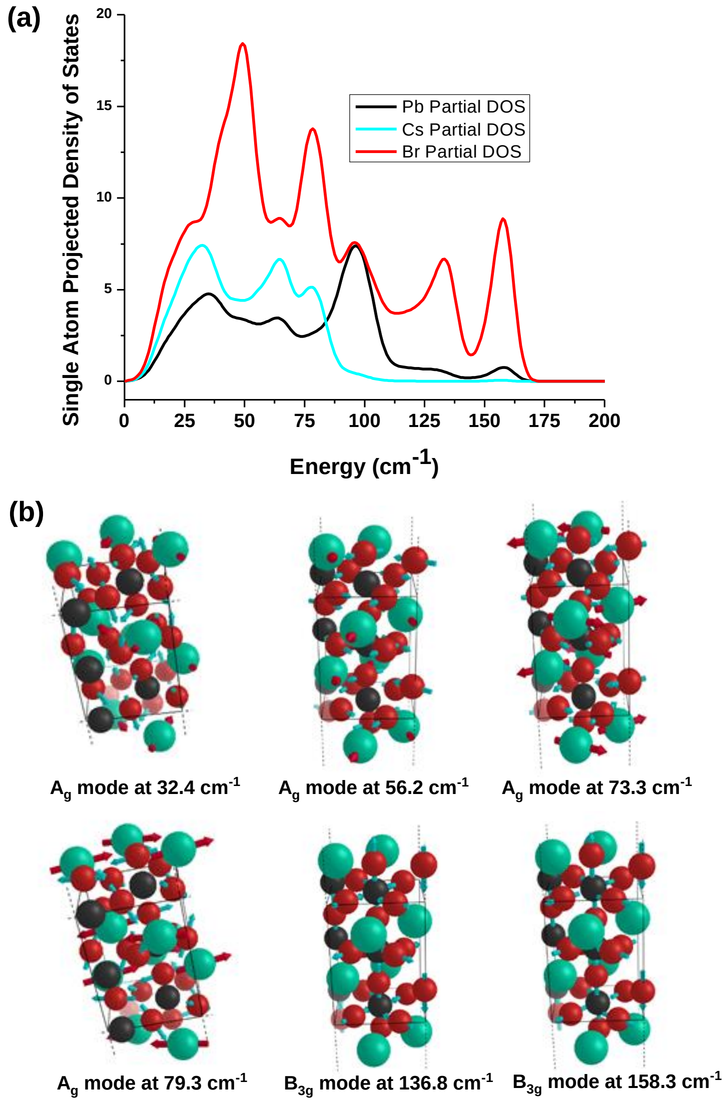

Fig. S3. (a) Partial phonon density of states derived from DFT simulations showing the Pb, Cs, and Br site projected components. (b) Selected Raman active phonon modes of $CsPbBr_3$ (see Table S1) indicating the motion of Cs (green), Br (red), and Pb (black) atoms.

Table S1 Calculated Phonon Modes (Raman Modes Labeled)*

Label	E (cm^{-1})	Atomic Motion in Raman Active Mode
Au	21.7	
B1u	25.5	
B2u	26.5	
B3u	29.3	
Au	30.2	
Ag	32.4	Shear motion of layers containing Cs and Br
B2g	34.7	Shear motion of layers containing Cs and Br
Au	35.5	
Ag	36.3	Out of phase Breathing motion of Br shell about Cs
B1u	36.5	
B2u	37.4	
B3u	38.3	
Au	39.1	
B2g	41.8	Complex Cs and Br motion
Ag	41.9	Complex Cs and Br motion
B1g	43.1	Motion of subset of Br atoms only
B1u	43.5	
B3u	46.0	
B3g	46.1	complex Cs and Br motion
B2g	47.6	Complex Cs and Br motion
B2u	48.4	
B1g	50.3	Complex Cs and Br motion
B3u	51.1	
B3g	51.9	Complex motion of Cs and subset of Br
B1u	52.3	
Ag	56.2	Complex Cs and Br motion
B2g	61.1	Complex Cs and Br motion
Au	63.1	
B3u	65.4	
B1u	65.6	
B2u	67.0	
Ag	73.3	Complex Cs and Br motion
B1u	75.4	
B3u	75.5	
B1g	75.6	Complex Cs and Br motion
B2g	75.8	
Ag	79.3	Complex Cs and Br motion
B3g	80.4	Complex Cs and Br motion
B2g	84.8	Complex Cs and Br motion
B1u	88.7	
Au	89.4	
B2u	91.8	
B3u	92.8	
B3u	93.5	
B2u	93.9	
Au	95.5	
B2u	96.7	
B1u	97.1	
Au	100.3	
B1u	100.4	
B3u	105.0	
B1g	134.3	Out of phase Breathing mode of $PbBr_6$ Unit
Ag	134.8	Out of phase Breathing mode of $PbBr_6$ Unit
B3g	136.8	Out of phase Breathing mode of $PbBr_6$ Unit
B2g	152.8	In-phase breathing mode of $PbBr_6$ Unit
B1g	153.4	In-phase breathing mode of $PbBr_6$ Unit
B3g	158.3	In-phase breathing mode of $PbBr_6$ Unit

*Raman Active Modes = 7Ag+ 5B1g +7B2g+5B3g

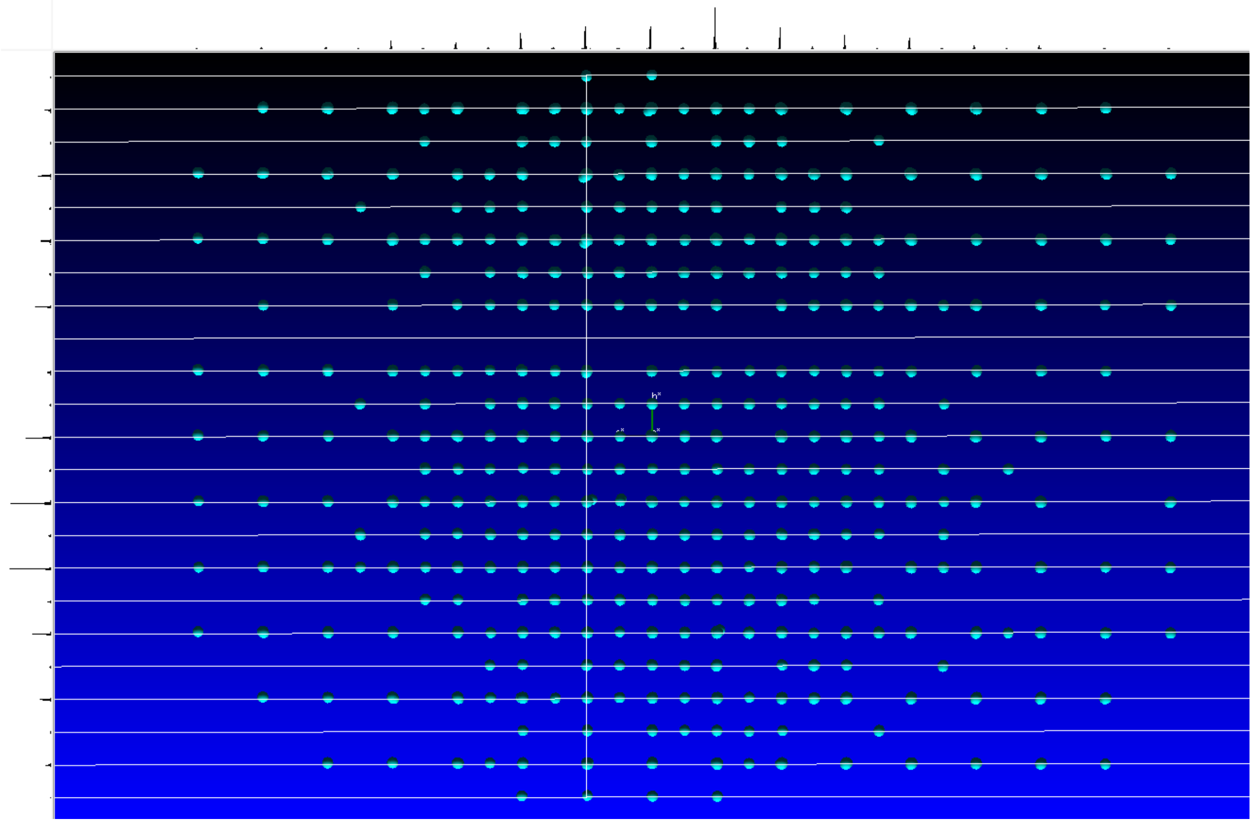

Fig. S4. View of reciprocal lattice points for data measured at 330 K.

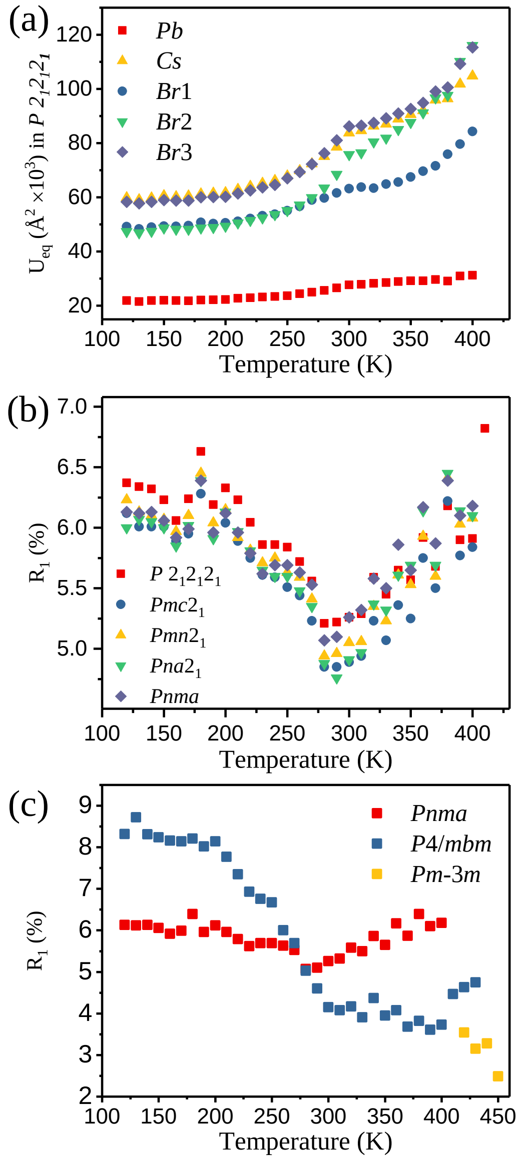

Fig. S5. (a) Temperature-dependent equivalent isotropic atomic displacement parameters (Å^2×10^3) from single-crystal data for $CsPbBr_3$ in the $P2_12_12_1$ space group. U_{eq} is defined as 1/3 of the trace of the orthogonalized U_{IJ} tensor. (b) The R_1 parameters of all possible orthorhombic space group based on the cell dimension $\sim\sqrt{2}\,a_P\;x\;\sqrt{2}\,a_P\;x\;2\,a_P$. Note that the R_1 values of the space groups are very close. However, systematic violations must also be examined (Table S2). (c) The R_1 based on the space groups in the literature. The unite cell for *Pnma* is $\sim\sqrt{2}\,a_P\times\sqrt{2}\,a_P\times 2\,a_P$, *P4/mbm* is $\sim\sqrt{2}\,a_P\times\sqrt{2}\,a_P\times a_P$, and $\sim a_P\times a_P\times a_P$ for *Pm*-3*m* space group.

Table S2-A. Calculated Atomic Displacements of *Im*-3 structure at 450 K Compared to *Pm*-3*m* Structure

Pm-3*m* Structure at 450 K				Transform *Pm*-3*m* to *Im*-3					*Im*-3 Structure at 450 K				Atomic Displacements (Å)
	x	y	z		x	y	z			x	y	z	
Pb1	0.5	0.5	0.5	Pb1	0.75	0.25	0.25	Compare	Pb1	0.75	0.25	0.25	0.0000
Cs1	0	0	0	Cs1	0.5	0.5	0.5	→	Cs1	0.5	0.5	0.5	0.0000
Br1	0	0.5	0.5	Cs2	0.5	0	0.5		Cs2	0.5	0	0.5	0.0000
				Br1	0.5	0.25	0.25		Br1	0.5	0.24328	0.25652	0.1100

*Both structures were solved from the same data set.

Table S2-B. Calculated Atomic Displacements of $P2_1/m$ structure at 380 K Compared to $P4/mbm$ Structure

*P*4/*mbm* Structure at 380 K				Transform *P*4/*mbm* to $P2_1/m$					$P2_1/m$ Structure at 380 K				Atomic Displacements (Å)
	x	y	z		x	y	z			x	y	z	
Pb1	0.5	0.5	0.5	Pb1	0.5	0	0		Pb1	0.5	0	0	0.0000
Cs1	1	0.5	0	Pb2	0	0	0	Compare	Pb2	0	0	0	0.0000
Br1	0.79195	0.70805	0.5	Pb3	0	0	0.5	→	Pb3	0	0	0.5	0.0000
Br2	0.5	0.5	0	Pb4	0.5	0	0.5		Pb4	0.5	0	0.5	0.0000
				Cs1	0.75	0.25	0.25		Cs1	0.76111	0.25	0.24104	0.1666
				Cs2	0.75	0.25	0.75		Cs2	0.75828	0.25	0.73748	0.1752
				Cs3	0.25	0.25	0.75		Cs3	0.26259	0.25	0.74357	0.1650
				Cs4	0.25	0.25	0.25		Cs4	0.25724	0.25	0.23861	0.1575
				Br1	0.45805	0	0.25		Br1	0.4567	-0.01829	0.24972	0.2160
				Br2	-0.04195	0	0.75		Br2	-0.04335	-0.01855	0.74967	0.2191
				Br3	0.75	0	0.45805		Br3	0.74946	0.0188	0.45626	0.2225
				Br4	0.5	0.25	0.5		Br4	0.47942	0.25	0.48303	0.3110
				Br5	0.75	0	0.04195		Br5	0.75044	-0.01753	0.04391	0.2078
				Br6	0	0.25	0		Br6	-0.02017	0.25	-0.01684	0.3063
				Br7	0.5	0.25	0		Br7	0.51575	0.25	0.01976	0.2946
				Br8	0	0.25	0.5		Br8	0.01827	0.25	0.52039	0.3192

*Both structures were solved from the same data set.

Table S2-C. Calculated Atomic Displacements of *Pm* structure at 250 K Compared to *Pnma* Structure

Pnma Structure at 250 K				Transform *Pnma* to *Pm*					*Pm* Structure at 250 K				Atomic Displacements (Å)
	x	y	z		x	y	z			x	y	z	
Pb1	0	0.5	0.5	Pb1	0.25	0.25	0.75		Pb1	0.25399	0.25016	0.75428	0.0112
Cs1	0.53865	0.25	0.49024	Pb2	0.75	0.25	0.75		Pb2	0.75409	0.25013	0.75424	0.0119
Br1	0.2956	0.47265	0.2966	Pb3	0.75	0.25	0.25		Pb3	0.75403	0.25019	0.25427	0.0115
Br2	-0.00421	0.25	0.55377	Pb4	0.25	0.25	0.25		Pb4	0.25413	0.25015	0.25428	0.0116
				Cs1	0.97579	0.0000	0.48555		Cs1	0.98282	0	0.4879	0.0500
				Cs2	0.47580	0.0000	0.98556		Cs2	0.48154	0	0.98541	0.0661
				Cs3	0.98555	0.0000	0.97579		Cs3	0.98882	0	0.97364	0.0858
				Cs4	0.48556	0.0000	0.47579		Cs4	0.48949	0	0.47423	0.0787
				Cs5	0.51445	0.5000	0.52420		Cs5	0.51748	0.5	0.51899	0.1216
				Cs6	0.01444	0.5000	0.02421		Cs6	0.01793	0.5	0.0239	0.0643
				Cs7	0.52421	0.5000	0.01445		Cs7	0.52909	0.5	0.01639	0.0399
				Cs8	0.02421	0.5000	0.51444		Cs8	0.02872	0.5	0.51619	0.0410
				Br1	0.29610	0.27735	0.49950	Compare	Br1	0.29878	0.27653	0.50528	0.0173
				Br2	0.22101	0.50000	0.77478	→	Br2	0.22567	0.5	0.78773	0.0906
				Br3	0.72101	0.50000	0.27478		Br3	0.72741	0.5	0.28772	0.0951
				Br4	0.79610	0.27735	-0.00050		Br4	0.79892	0.27659	0.00504	0.0148
				Br5	-0.00050	0.27735	0.29610		Br5	0.00244	0.27735	0.30208	0.0132
				Br6	0.72522	0.00000	0.77899		Br6	0.73088	0	0.78717	0.0410
				Br7	0.50050	0.22265	0.20390		Br7	0.50355	0.22181	0.21042	0.0200
				Br8	0.70390	0.22265	0.50050		Br8	0.70625	0.22159	0.50595	0.0210
				Br9	0.20390	0.22265	0.00050		Br9	0.20649	0.22186	0.00621	0.0177
				Br10	0.49950	0.27735	0.79610		Br10	0.50246	0.27802	0.80184	0.0138
				Br11	0.22522	0.00000	0.27899		Br11	0.23105	0	0.28739	0.0442
				Br12	0.00050	0.22265	0.70390		Br12	0.00334	0.22343	0.70941	0.0147
				Br13	0.27478	0.50000	0.22101		Br13	0.27915	0.5	0.23105	0.0566
				Br14	0.77478	0.50000	0.72101		Br14	0.77877	0.5	0.73025	0.0469
				Br15	0.27899	0.00000	0.72522		Br15	0.28251	0	0.73641	0.0695
				Br16	0.77899	0.00000	0.22522		Br16	0.78335	0	0.23748	0.0823

*Both structures were solved from the same data set.

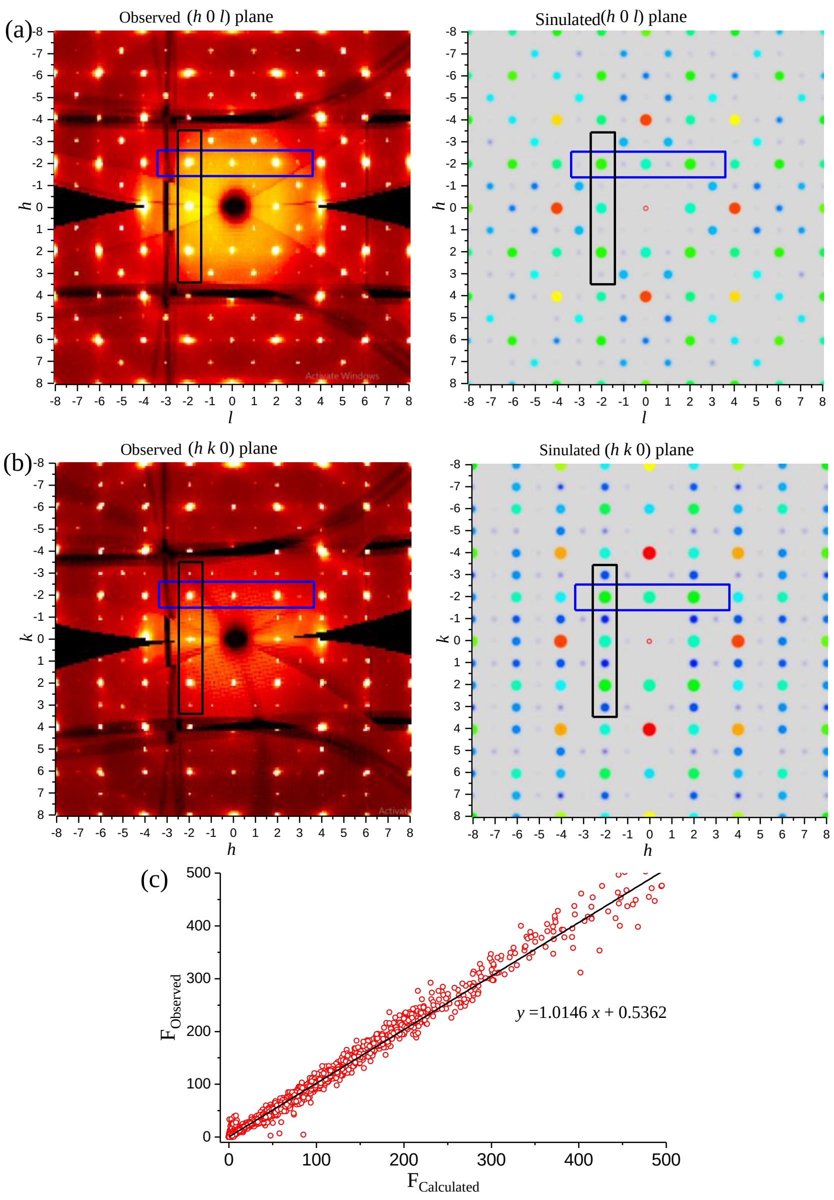

Fig. S6. Experimental reciprocal space precession images at 380 K for the (a) (*h* 0 *l*) plane and (b) (*h k* 0) plane indexed with a $2a_P \times 2a_P \times 2a_P$ cell compared to the simulated pattern using the $P2_1/m$ crystal structure solution ($2a_P \times 2a_P \times 2a_P$). The *b* axis is the long axis. Observe the qualitative similarity in the weak peaks representing the doubled cell relative to the simple cubic cell ($a_P \times a_P \times a_P$). Rectangles with the same color cover equivalent regions in measured and simulated images. In the calculation, the high-intensity peaks are in the red region of the color spectrum and the low-intensity peaks are on the blue end of the spectrum. The size of the peaks shown also indicates their intensities. (c) The $F_{Observed}$ vs $F_{Calculated}$ is fitted by a linear function at 380 K.

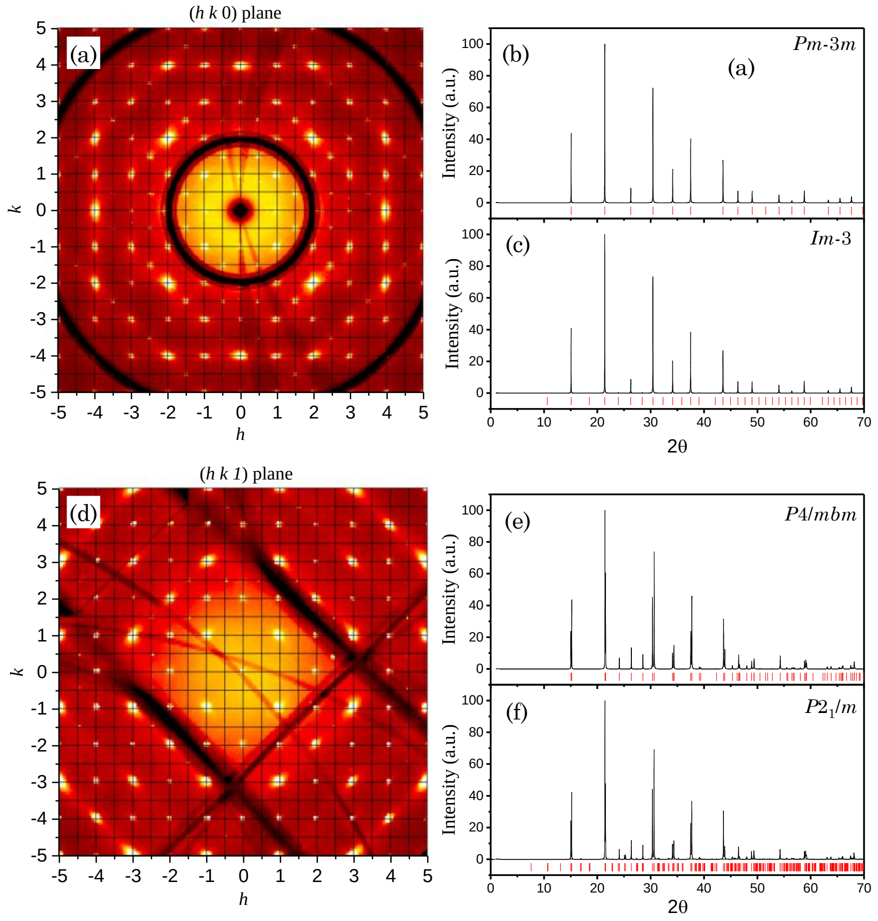

Fig. S7. (a) Single-crystal X-ray diffraction reciprocal space images of the (*h k* 0) planes of $CsPbBr_3$ at 450 K. The (*h k l*) grid corresponds to the previously reported *Pm*-3*m* space group with a lattice constant *a* = 5.87 Å. Diffraction spots with half-integer values are observed, indicating that the correct lattice constant should be doubled. The simulated powder diffraction patterns of the *Pm*-3*m* and the newly proposed *Im*-3 space group based on the solved structure from single-crystal diffraction refinement are given in panels (b) and (c), respectively. (d) Single-crystal X-ray diffraction reciprocal space images of the (*h k* l) planes of $CsPbBr_3$ at 360 K. The grid corresponds to the previously reported *P*4/*mbm* space group with unit cell dimension: $\sqrt{2}\,a_P \times \sqrt{2}\,a_P \times a_P$. Note the presence of half-integer peaks. The simulated powder diffraction patterns of *P*4/*mbm* and the newly proposed $P2_1/m$ space group based on the solution of the single-crystal diffraction refinement are given in panels (e) and (f), respectively. The wavelength for the simulated powder diffraction patterns is 1.54059 Å (Cu-Kα). Powder diffraction measurements are not adequate to distinguish between the *P*4/*mbm* and $P2_1/m$ space groups in the 360 K data and between the *Pm*-3*m* and *Im*-3 space groups in the 450 K data. We also note that above 360 K, the *Im*-3*m* and *Im*-3 space groups have similar R_1 parameters. However, the Cs ADPs of *Im*-3*m* are highly anomalous (dramatically reduced in size with temperature increase). The anomalous behavior is due to the presence of distortions in $CsPbBr_3$ not supported by the high symmetry *Im*-3*m* space group.

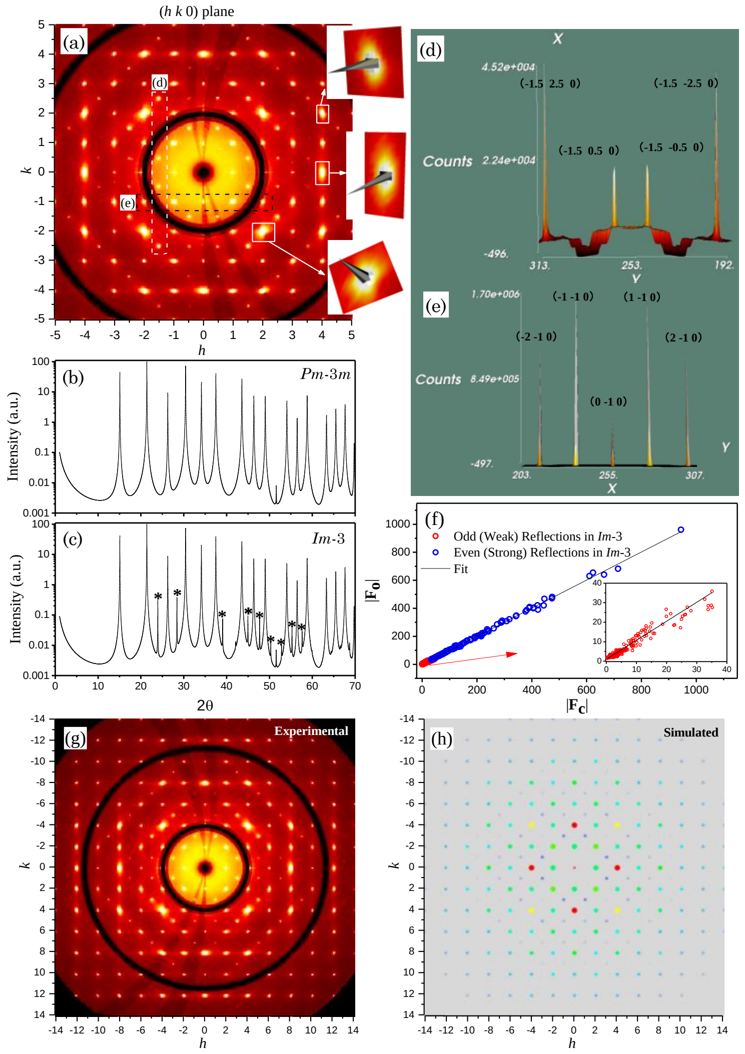

Fig. S8. (a) Single-crystal X-ray diffraction reciprocal space images of the (*h k* 0) planes of $CsPbBr_3$ at 450 K. The (*h k l*) grid corresponds to the *Pm*-3*m* (#221) space group with unit cell dimension: $\sim a_P \times a_P \times a_P$ where *a* = 5.87 Å. However, the presence of half-integer peaks indicates the *Pm*-3*m* simple cell is incorrect. The inset shows the 3D intensity of some selected reflections with an asymmetric diffuse scattering background. (b) Simulated *Pm*-3*m* powder XRD pattern with cell dimension: $\sim a_P \times a_P \times a_P$. (c) Simulated *Im*-3 (#204) powder XRD pattern with cell dimension: $\sim 2a_P \times 2a_P \times 2a_P$. The intensity (y-axis) is displayed on a log scale. The additional features in the spectra are indicated with asterisks (*) symbols corresponds to the half-integer peaks in reciprocal lattice shown in panel (a). In the reciprocal space images, an examination of the intensities of half-integer reflections, (-1.5 *k* 0) is shown in panel (d), and the integer reflections, (*h* -1 0) is in panel (e), reveals the intensity of half-integer peaks are ~10^2 times weaker than the integer peak intensity but ~10 times stronger than the intensity of the background. Returning to the powder diffraction simulations [panel (c)], it is observed that the weak peaks (*) are of the same level relative to the main peaks as what is seen in the reciprocal space images (d) for the single-crystal measurements. Hence fitting powder data to the *Pm-3m* structure will not be strongly influenced by the exclusion of these additional peaks. Consequently, powder diffraction can not be used to ascertain the space group. A plot of $|F_o|$ (observed) vs. $|F_c|$ (calculated) parameters at 450 K for the strong (even-integer) and the weak satellite (odd-integer) reflections in *Im*-3 space group with a linear fit (black line). The inset is the same plot with data for the odd-integer reflections. (g) Experimental reciprocal space image at 450 K for the (*h k* 0) plane compared with the simulated pattern (h) for the *Im*-3 structure ($2a_P \times 2a_P \times 2a_P$). In the calculation, the high-intensity peaks are in the red region of the color spectrum and the low-intensity peaks are on the blue end of the spectrum. The size of the peaks shown also indicates their intensities.

Table S3. Refined Structural Parameters Utilizing the Weak Reflections Exclusively at 450 K in *Im*-3 Space Group

**Atoms**	**x**	**y**	**z**	$\mathbf{U_{eq}}$ (Å^3×10^3)
Pb1	7500	2500	2500	37(3)
Cs1	5000	5000	5000	75(5)
Cs2	5000	0	5000	75(5)
Br1	5000	2491(6)	2509(6)	81(4)

Space Group: *Im*-3
a = 11.7444 (3) Å, Dx = 4.755 g/cm^3
Measurement Temperature: 450 K
Crystal Dimensions (diameter): ~ 50 μm
Wavelength: 0.41328 Å
2θ range for data collection: 2.852° to 40.228°
Index ranges: $-18 \leq h \leq 18$, $-18 \leq k \leq 18$, $-18 \leq l \leq 13$
Reflections collected: 26205
Independent reflections: 513
Number of fitting parameters: 7
Largest diff. peak/hole: 0.38/-0.39 e/Å^3
R_1 = 50.18 %, wR_2 = 71.60 %, Goodness of Fit = 2.112

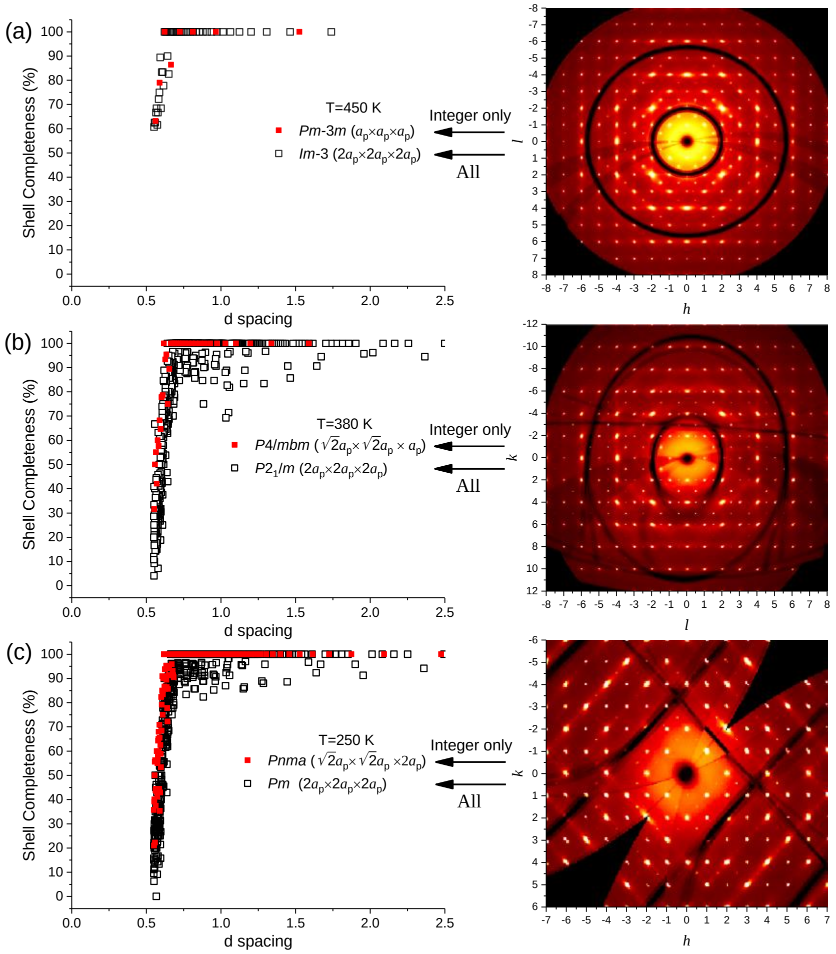

Fig. S9. The completeness as a function of d spacing is given for both the previously reported models and new models. Reflections are binned in d space using 20 bins for all models. This gives a representative sample of the density of reflections. Note that not all reflections are captured on this coarse grid. The reciprocal lattice precession images are also present to better clarify where are the additional reflections occur in the raw data. The (*h k l*) grids in these images are based on the old unit cell dimensions. The shell completeness of the old model is presented as the solid red square symbols and the new model as open square symbols. Note that, for the old models, mainly strong reflections (integer reflections) in the full data set are utilized. While for the new model, all the reflections (both integer and half-integer reflections) are captured and utilized in the structural refinement. The half-integer reflections are not fitted in the old models. In panel (c), it's not surprising that some weak reflections appear at low temperatures which are difficult to be captured completely. In our structural solutions, the overall completeness for the $P2_1/m$ and *Pm* solution is >97% while for *Im*-3 is >99%. The completeness of old models is >99% since they require only a subset (dominant reflections only) of the measured reflections.

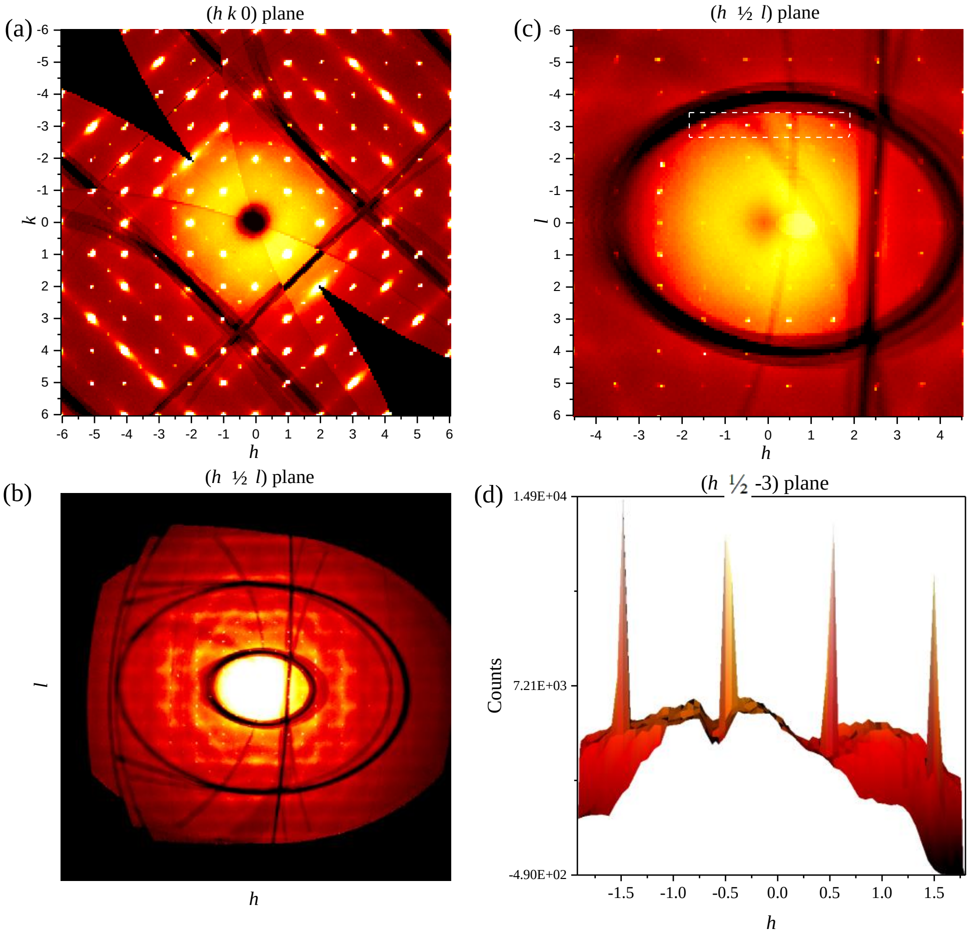

Fig. S10. (a) Single-crystal X-ray diffraction reciprocal space image of the (*h k* 0) plane at 200 K. The grid corresponds to the previously reported orthorhombic (*Pnma*) unit cell dimension ~ $\sqrt{2}\, a_P \times \sqrt{2}\, a_P \times 2a_P$. The half-integer reflections can be observed along the diagonal. (b) The reciprocal space image of the (*h* ½ *l*) plane shows the half-integer reflections, and the expanded image is given in (c). (d) The intensity map for the (*h* ½ -3) line corresponds to the selected region in panel (c). We found that these reflections are weak (~ 10 times the background intensity). Careful considerations showed that all these previously unfitted weak reflections can indeed be indexed on a primitive monoclinic supercell with *a* =11.6126(6) Å, *b* =11.7344(6) Å, *c* =11.6156(5) Å, and β =89.1610(10). Considering all observed reflections, the space group can no longer be taken as *Pnma* space group. The true space group is *Pm* with unit cell dimensions ~ $2a_P \times 2a_P \times 2a_P$.

Table S4-A. Number of Systematic Absence Violations*

Temperature (K)	Space group				
	$P2_12_12_1$	$Pmc2_1$	$Pmn2_1$	$Pna2_1$	*Pnma*
100	7	90	47	137	137
110	6	98	52	150	150
120	6	79	46	125	125
130	5	97	53	150	150
140	5	77	46	123	123
150	6	85	58	143	143
160	6	82	54	136	136
170	8	90	56	146	146
180	6	71	46	117	117
190	3	90	46	136	136
200	4	73	41	114	114
210	7	77	42	119	119
220	5	69	40	109	109
230	6	85	50	135	135
240	4	67	48	115	115
250	4	99	53	152	152
260	7	133	91	224	224
270	9	136	95	231	231
280	6	148	86	234	234
290	8	190	121	311	311
300	11	228	150	378	378
310	7	244	151	395	395
320	10	220	141	361	361
330	8	228	146	374	374
340	9	228	160	388	388
350	6	201	143	344	344
360	7	140	129	269	269

* Above 360 K, R_1 of the orthorhombic space group is larger than 10% and is not presented in the table.

Table S4-B. Sample Peaks Violating Conditions of the *Pnma* Space Group (250 K)*

(*h k l*)	F_0^2	$\sigma(F_0^2)$	
(0 -7 2)	164.08	14.6	observed (0 *k l*) reflections,
(0 7 -2)	163.48	14.	*k* +l not even
(0 -7 -2)	109.39	10.5	
(0 7 2)	104.99	10.1	
(0 -11 -2)	73.29	7.8	
(0 11 -2)	43.9	4	
(0 -11 -2)	73.29	7.8	
(0 9 -2)	5.9	0.9	
(0 -9 -2)	5.8	1.3	
(1 -7 0)	379.96	34.2	observed (*h k* 0) reflections,
(-1 7 0)	373.66	34.4	*h* not even
(-1 -7 0)	414.86	34.8	
(1 7 0)	401.56	34.7	
(1 -5 0)	266.47	22	
(-1 5 0)	251.47	21.9	
(-1 -5 0)	202.78	17.4	
(1 5 0)	180.58	17.4	
(3 -9 0)	128.09	11.4	
(-3 9 0)	109.69	11.5	
(1 11 0)	81.69	7.1	
(-1 -11 0)	56.89	6.8	
(1 -9 0)	76.89	6.7	
(-1 9 0)	68.19	6.6	
(1 9 0)	72.49	6.6	
(-1 -9 0)	63.49	6.4	
(-3 5 0)	22.5	2.3	
(3 -5 0)	22.3	2.3	

* The largest peak observed with respect to the the *Pnma* space group is the (4 0 0) reflections (scaled $|F_0|^2$ =10,000 and $\sigma(|F_0|^2)$ = 830). The intensities of the extinction violating peaks in *Pnma* are 10^2 times weaker than the main peak.

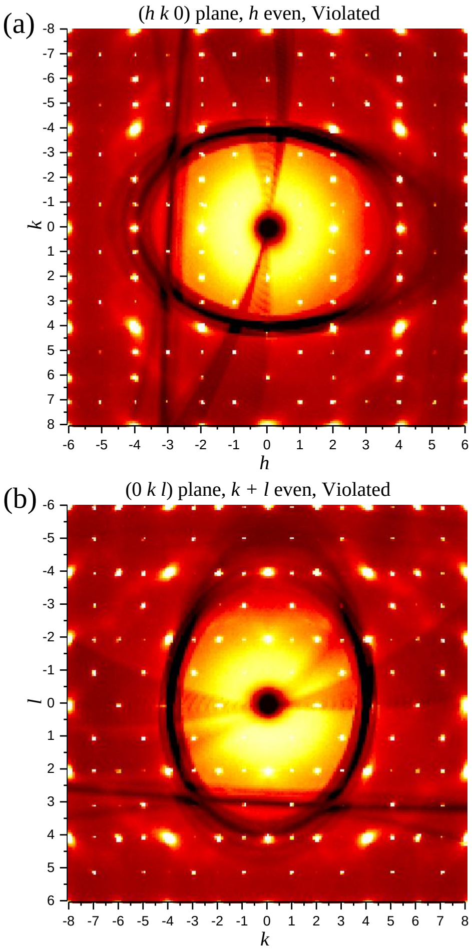

Fig. S11. Single-crystal X-ray diffraction reciprocal space precession images of (a) (*h k* 0) plane and (b) (0 *k l*) planes of $CsPbBr_3$ at 250 K. The grid corresponds to the previously reported *Pnma* space group with unit cell dimension: $\sim\sqrt{2}\, a_P \times \sqrt{2}\, a_P \times\ 2a_P$ (*a* =8.2646 Å, *b* =11.7416 Å, *c* =8.1707 Å). Two of the *Pnma* space group reflection conditions are 0 *k l*: *k*+*l* =2n and *h k* 0: *h* =2n. Both conditions are violated by the measured data (Also see Table S2-B).

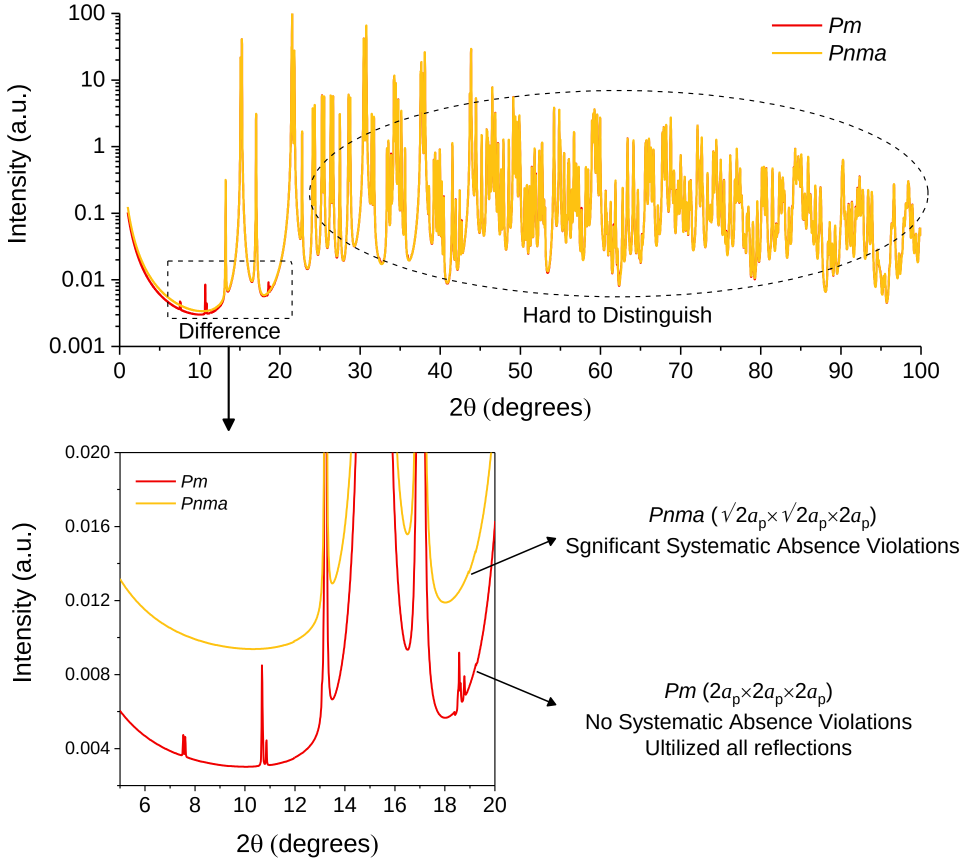

Fig. S12-A. The simulated powder diffraction patterns of the low-temperature models: previously reported *Pnma* structure and the newly assigned *Pm* structure. The wavelength for the simulated powder diffraction patterns is 1.54059 Å (Cu-Kα). The y-axis is the intensity on the log scale. The unit cell dimension of orthorhombic models is $\sqrt{2}\ a_P \times \sqrt{2}\ a_P \times a_P$, while the monoclinic *Pm* model is ~ $2a_P \times 2a_P \times 2a_P$. It is very difficult to distinguish the difference between the two models at a high 2θ angle since multiple peaks merge. Note that the additional features that appear in the *Pm* model are of low intensity (10^4 times weaker than main peaks). To refine the structures, all reflections are utilized in the *Pm* model and reveals the real structure is polar. The orthorhombic *Pnma* model has non-indexed half-integer reflections which are the additional features seen in the *Pm* powder diffraction pattern.

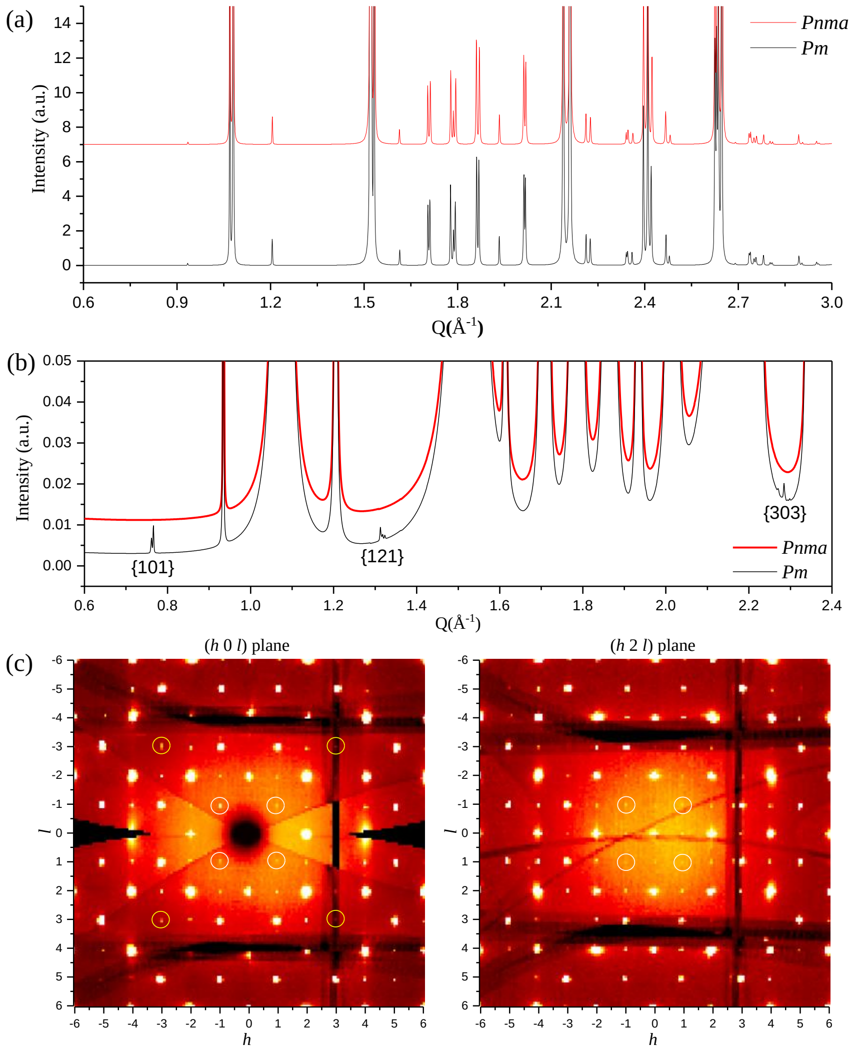

Fig. S12-B. (a) The simulated powder diffraction patterns of the low-temperature models: previously reported *Pnma* structure and the newly assigned *Pm* structure. The wavelength for the simulated powder diffraction patterns is 1.54059 Å (Cu-Kα). The y-axis is the intensity on the linear scale. All dominant peaks match up. (b) Additional reflections in the calculated *Pm* powder pattern compared with the pattern of the *Pnma* space group. The additional reflections in the *Pm* space group are labeled. (c) The corresponding (*h k l*) peaks of the additional features in the *Pm* space group are observed in single-crystal diffraction data (at 280 K). Note that the *b* axis is the long axis in the *Pm* structure. The circled reflection peaks are the additional peaks observed in the *Pm* structure which are labeled in Panel (b).

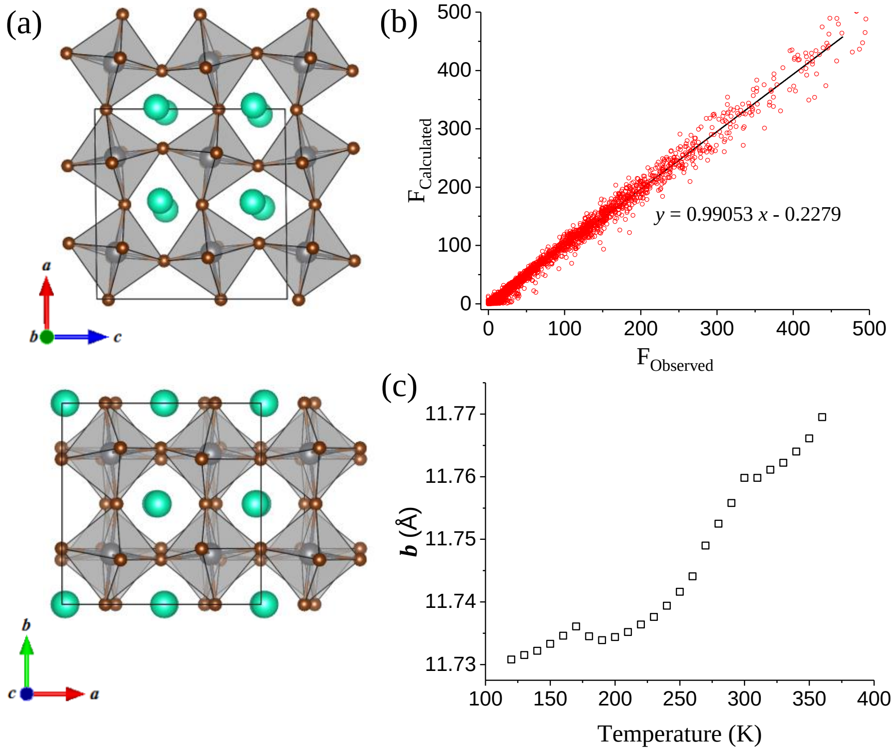

Fig. S13-A. (a) Solved structures from single-crystal X-ray diffraction measurements in the *Pm* space group. (b) The $F_{measured}$ vs $F_{calculated}$ is fitted by a linear function (at 120 K). (c) The lattice parameters *b* as a function of temperature.

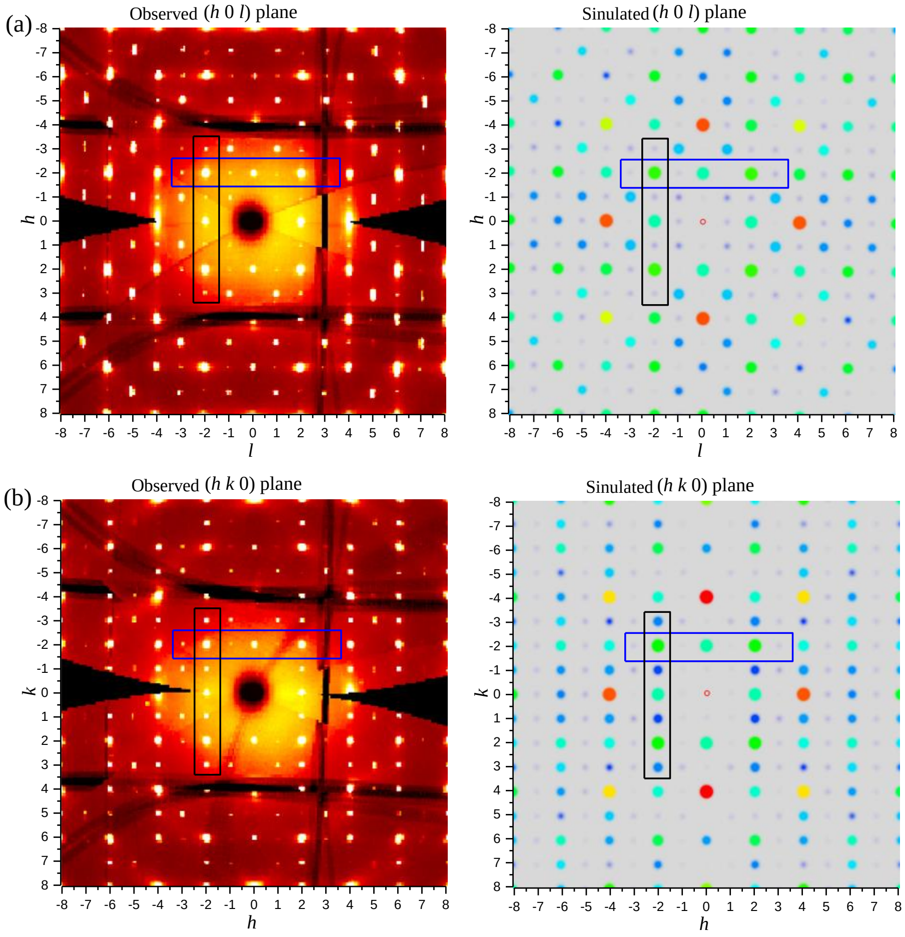

Fig. S13-B. Experimental reciprocal space precession images (120 K) for the (a) (h 0 l) plane and (b) (h k 0) plane indexed with a $2a_P \times 2a_P \times 2a_P$ cell compared to the simulated pattern using the Pm crystal structure solution ($2a_P \times 2a_P \times 2a_P$). The b axis is the long axis. Observe the qualitative similarity in the weak peaks representing the doubled cell relative to the simple cubic cell ($a_P \times a_P \times a_P$). Rectangles with the same color cover equivalent regions in measured and simulated images. In the calculation, the high-intensity peaks are in the red region of the color spectrum and the low-intensity peaks are on the blue end of the spectrum. The size of the peaks shown also indicates their intensities.

Table S5. Structural Parameters from $CsPbBr_3$ at 450 K in *Im*-3 Space Group

**Atoms**	**x**	**y**	**z**	$\mathbf{U_{eq}}$ (Å^3×10^3)		
Pb1	7500	2500	2500	37.0(3)		
Cs1	5000	5000	5000	117.1(12)		
Cs2	5000	0	5000	118.5(14)		
Br1	5000	2432.8(16)	2565.2(16)	158.4(13)		
U_{ij} (Pb1)	37.0(3)	37.0(3)	37.0(3)	-0.02(5)	-0.02(5)	-0.02(5)
U_{ij} (Cs1)	118.8(15)	102.9(13)	129.5(18)	0	0	0
U_{ij} (Cs2)	118.5(14)	118.5(14)	118.5(14)	0	0	0
U_{ij} (Br1)	225(4)	219(4)	31.3(6)	0	0	11.1(12)

Space Group: *Im*-3
a = 11.7444 (3) Å, Dx = 4.755 g/cm^3
Measurement Temperature: 450 K
Crystal Dimensions (diameter): ~50 μm
Wavelength: 0.41328 Å
2θ range for data collection: 2.852° to 44.138°
Index ranges: -18 ≤ h ≤ 18, -18 ≤ k ≤ 18, -18 ≤ l ≤ 13
Reflections collected: 35245
EXTI extinction parameter: 0.0107(11)
Independent reflections: 863
Number of fitting parameters: 14
Largest diff. peak/hole: 1.32(Cs2)/-1.20(Br1) e Å^{-3}
R_1 = 3.30 %, wR_2 = 8.40 %, Goodness of Fit = 1.012

*Atomic displacement parameters U_{ij} (Å^2×10^3) are in the order U_{11}, U_{22}, U_{33}, U_{23}, U_{13}, U_{12}

Table S6. Structural Parameters from $CsPbBr_3$ at 380 K in $P2_1/m$ Space Group

**Atoms**	**x**	**y**	**z**	$\mathbf{U_{eq}}$ ($Å^3 \times 10^3$)		
Pb1	5000	0	0	28.35(19)		
Pb2	0	0	0	28.42(19)		
Pb3	0	0	5000	28.29(19)		
Pb3	5000	0	5000	28.44(19)		
Cs1	7611.1(19)	2500	2410.4(11)	92.1(6)		
Cs2	7582.8(12)	2500	7374.8(18)	87.1(5)		
Cs3	2625.9(2)	2500	7435.7(11)	91.0(6)		
Cs4	2572.4(13)	2500	2386.1(2)	96.2(6)		
Br1	4567(16)	-182.9(14)	2497.2(10)	80.7(4)		
Br2	-433.5(16)	-185.5(14)	7496.7(10)	80.1(4)		
Br3	7494.6(10)	188(14)	4562.6(14)	79.3(4)		
Br4	4794.2(3)	2500	4830.3(3)	92.9(8)		
Br5	7504.4(11)	-175.3(14)	439.1(15)	80.3(4)		
Br6	-201.7(3)	2500	-168.4(3)	97.1(9)		
Br7	5157.5(3)	2500	197.6(3)	97.2(9)		
Br8	182.7(3)	2500	5203.9(3)	91.1(8)		
U_{ij} (Pb1)	30.6(3)	26.4(3)	28.1(3)	0.83(8)	1.5(3)	-0.15(8)
U_{ij} (Pb2)	31.1(3)	26.9(3)	27.3(3)	0.06(8)	1.6(3)	-0.73(9)
U_{ij} (Pb3)	30.7(3)	26.6(3)	27.6(3)	0.77(8)	1.6(3)	-0.10(8)
U_{ij} (Pb4)	30.9(3)	26.6(3)	27.8(3)	0.15(8)	1.6(3)	-0.76(8)
U_{ij} (Cs1)	149.7(18)	61.3(7)	65.2(7)	0	-1.1(10)	0
U_{ij} (Cs2)	68.8(8)	66.0(8)	126.6(13)	0	-8.1(9)	0
U_{ij} (Cs3)	146.9(17)	64.6(8)	61.6(6)	0	-6.7(9)	0
U_{ij} (Cs4)	69.1(9)	62.7(8)	156.7(17)	0	-17.2(10)	0
U_{ij} (Br1)	97.4(10)	118.5(10)	26.1(4)	0.7(5)	2.5(5)	-11.8(8)
U_{ij} (Br2)	97.3(10)	117.5(10)	25.5(4)	-3.1(5)	2.4(5)	-10.9(8)
U_{ij} (Br3)	27.8(4)	115.8(10)	94.3(9)	15.1(7)	2.4(5)	2.2(5)
U_{ij} (Br4)	134(2)	27.2(6)	117.1(18)	0	-0.8(15)	0
U_{ij} (Br5)	27.6(5)	118.9(10)	94.4(10)	8.6(7)	2.1(5)	-0.1(5)
U_{ij} (Br6)	149(2)	27.5(6)	114.8(18)	0	1.3(16)	0
U_{ij} (Br7)	125(2)	24.7(6)	142(2)	0	3.2(16)	0
U_{ij} (Br8)	112.6(17)	26.6(6)	134.1(19)	0	5.6(15)	0

Space Group: $P2_1/m$
a = 11.6630(4) Å, b = 11.7796(5) Å, c = 11.6664(5) Å, β = 90.0570(10)°, Dx = 4.806 g/cm^3
Measurement Temperature: 380 K
Crystal Dimensions (diameter): ~50 µm
Wavelength: 0.41328 Å
2θ range for data collection: 2.01° to 44.144°
Index ranges: $-18 \leq h \leq 13$, $-18 \leq k \leq 18$, $-18 \leq l \leq 18$
Reflections collected: 68421
Twin law: 1 0 0 0 -1 0 0 0 -1 2
BASF parameter: 0.265(4)
EXTI extinction parameter: 0.0118(12)
Independent reflections: 7966
Number of fitting parameters: 111
Largest diff. peak/hole: 4.89(Pb4)/-6.8(Cs4) e Å^{-3}
R_1 = 7.22 %, wR_2 = 32.47 %, Goodness of Fit = 1.006

*Atomic displacement parameters U_{ij} ($Å^2 \times 10^3$) are in the order U_{11}, U_{22}, U_{33}, U_{23}, U_{13}, U_{12}.
** The Pseudomerohedry twin fraction components are 0.735(5) and 0.265(5).

Table S7. Structural Parameters from $CsPbBr_3$ at 340 K in $P2_1/m$ Space Group

**Atoms**	**x**	**y**	**z**	$\mathbf{U_{eq}}$ (Å^3×10^3)		
Pb1	5000	0	0	28.14(13)		
Pb2	0	0	0	28.13(13)		
Pb3	0	0	5000	28.09(13)		
Pb3	5000	0	5000	28.16(13)		
Cs1	7656.1(18)	2500	2383.2(10)	84.9(5)		
Cs2	7612.6(12)	2500	7325.3(16)	79.7(4)		
Cs3	2674.1(18)	2500	7403.9(10)	83.5(4)		
Cs4	2604.8(13)	2500	2332.6(18)	88.8(4)		
Br1	4559.7(13)	-232.7(12)	2497.5(8)	70.9(4)		
Br2	-441.1(13)	-235.7(12)	7498.3(8)	70.6(4)		
Br3	7497.3(10)	236.8(12)	4562.3(12)	69.9(3)		
Br4	4749.8(3)	2500	4789.6(2)	81.4(6)		
Br5	7501.4(11)	-227.3(12)	441.7(12)	70.5(3)		
Br6	-252.3(3)	2500	-215.9(2)	85.0(6)		
Br7	5195.8(2)	2500	238.1(3)	86.4(6)		
Br8	223.2(2)	2500	5246.9(2)	79.6(6)		
U_{ij} (Pb1)	30.8(2)	25.4(2)	28.2(2)	1.10(10)	1.04(16)	-0.21(8)
U_{ij} (Pb2)	31.0(2)	25.7(2)	27.6(2)	0.17(10)	1.16(16)	-1.18(8)
U_{ij} (Pb3)	30.9(2)	25.7(2)	27.7(2)	1.05(10)	1.20(16)	-0.13(8)
U_{ij} (Pb4)	31.0(2)	25.3(2)	28.2(2)	0.17(10)	1.02(16)	-0.94(8)
U_{ij} (Cs1)	139.5(14)	56.9(6)	58.3(5)	0	-3.3(7)	0
U_{ij} (Cs2)	63.9(8)	60.8(6)	114.2(10)	0	-4.2(7)	0
U_{ij} (Cs3)	130.4(13)	60.6(6)	59.5(6)	0	-6.6(7)	0
U_{ij} (Cs4)	64.5(9)	59.7(6)	142.0(13)	0	-14.3(8)	0
U_{ij} (Br1)	89.5(9)	97.5(8)	25.7(3)	1.7(4)	1.5(4)	-11.6(7)
U_{ij} (Br2)	90.9(9)	96.2(8)	24.5(3)	-1.3(4)	1.4(4)	-11.5(7)
U_{ij} (Br3)	28.9(5)	95.6(8)	85.2(7)	14.2(6)	2.0(4)	1.9(5)
U_{ij} (Br4)	121.2(16)	23.2(5)	99.7(13)	0	-8.0(12)	0
U_{ij} (Br5)	29.5(5)	98.1(8)	83.9(7)	9.3(6)	1.3(4)	-1.4(5)
U_{ij} (Br6)	134.1(18)	24.4(5)	96.6(12)	0	-9.0(13)	0
U_{ij} (Br7)	111.5(16)	21.0(5)	126.5(16)	0	-0.9(13)	0
U_{ij} (Br8)	97.3(14)	23.2(5)	118.2(15)	0	-1.0(12)	0

Space Group: $P2_1/m$
a = 11.6457(4) Å, b = 11.7640(4) Å, c = 11.6497(4) Å, β = 90.1520(10)°, Dx = 4.826 g/cm^3
Measurement Temperature: 340 K
Crystal Dimensions (diameter): ~50 μm
Wavelength: 0.41328 Å
2θ range for data collection: 2.012° to 44.134°
Index ranges: $-13 \leq h \leq 18$, $-18 \leq k \leq 18$, $-18 \leq l \leq 18$
Reflections collected: 70053
Twin law: 1 0 0 0 -1 0 0 0 -1 2
BASF parameter: 0.192(3)
EXTI extinction parameter: 0.0154(9)
Independent reflections: 7584
Number of fitting parameters: 111
Largest diff. peak/hole: 3.36(Cs2)/-5.06(Cs1) e Å^{-3}
R_1 = 6.10 %, wR_2 = 22.64 %, Goodness of Fit = 1.003

*Atomic displacement parameters U_{ij} (Å^2×10^3) are in the order U_{11}, U_{22}, U_{33}, U_{23}, U_{13}, U_{12}.
** The Pseudomerohedry twin fraction components are 0.808(3) and 0.192(3).

Table S8. Structural Parameters from $CsPbBr_3$ at 280 K in *Pm* Space Group

Atoms	x	y	z	U_{eq}	U_{11}	U_{22}	U_{33}	U_{23}	U_{13}	U_{12}
Pb1	2531.5(9)	2498.7(5)	7531.1(8)	26.44(18)	30.9(4)	22.6(3)	25.8(3)	-1.25(15)	1.2(2)	0.05(14)
Pb2	7531.3(9)	2499.1(5)	7527.8(8)	26.48(18)	30.8(4)	23.0(3)	25.6(3)	-0.22(15)	1.1(2)	1.03(14)
Pb3	7532.1(9)	2499.0(5)	2527.6(8)	26.47(18)	30.9(4)	23.0(3)	25.5(3)	-1.13(15)	1.1(2)	0.31(14)
Pb4	2531.2(9)	2498.8(5)	2531.1(8)	26.37(18)	30.8(4)	22.6(3)	25.7(3)	-0.14(15)	1.3(2)	1.25(14)
Cs1	10255(5)	0	4909(4)	71.5(14)	115(4)	50.1(11)	49.5(16)	0	-6.8(19)	0
Cs2	5251(5)	0	9905(4)	72.0(14)	118(4)	48.8(11)	48.9(15)	0	-5.9(19)	0
Cs3	10145(5)	0	9890(6)	85.7(18)	50(3)	50.8(13)	156(5)	0	-13(3)	0
Cs4	5170(5)	0	4846(6)	76.7(14)	50(3)	52.9(12)	127(4)	0	-4(2)	0
Cs5	4893(5)	5000	5285(5)	74.2(12)	74(3)	55.4(13)	94(3)	0	-8(2)	0
Cs6	-95(5)	5000	277(5)	71.3(11)	72(3)	56.2(12)	86(2)	0	-3.9(19)	0
Cs7	4821(6)	5000	167(5)	81.1(16)	117(5)	58.4(14)	68(2)	0	-3(2)	0
Cs8	-154(6)	5000	5189(5)	84.0(18)	136(6)	56.4(14)	59(2)	0	-5(2)	0
Br1	2967(5)	2239(3)	5013(4)	62.6(9)	79(3)	83.6(16)	25.2(8)	-1.8(11)	-1.4(12)	10.7(15)
Br2	2289(8)	5000	7239(8)	80(2)	87(5)	24.4(13)	129(6)	0	-14(4)	0
Br3	7280(7)	5000	2229(7)	73.8(19)	81(5)	26.5(12)	114(5)	0	-8(3)	0
Br4	7977(5)	2247(2)	9(4)	60.9(9)	75(3)	82.8(16)	24.5(8)	-0.5(11)	-0.8(11)	8.7(15)
Br5	30(5)	2771(2)	2959(4)	61.7(8)	28(2)	79.8(16)	78(2)	-12.8(13)	0.2(13)	-2.5(12)
Br6	7259(9)	0	7254(7)	84(2)	128(7)	27.9(14)	96(5)	0	-16(4)	0
Br7	5023(5)	2242(2)	2065(4)	61.7(8)	31(2)	80.8(16)	73(2)	-8.5(13)	2.8(14)	0.6(13)
Br8	7072(5)	2769(2)	5003(4)	63.8(10)	90(3)	80.1(15)	21.0(8)	1.5(10)	3.9(12)	13.2(15)
Br9	2062(5)	2758(2)	5(4)	63.2(10)	85(3)	82.3(15)	21.9(8)	0.5(10)	4.2(12)	8.7(15)
Br10	5027(5)	2754(2)	7969(4)	61.2(8)	29(2)	79.9(16)	75(2)	-7.8(13)	-0.7(13)	-0.1(12)
Br11	2261(9)	0	2267(8)	82(2)	115(7)	28.2(14)	104(5)	0	-11(4)	0
Br12	24(5)	2237(3)	7069(4)	62.6(8)	32(2)	82.8(16)	73(2)	-10.9(13)	3.2(14)	1.8(13)
Br13	2803(7)	5000	2735(6)	64.0(17)	104(5)	15.9(9)	72(3)	0	1(3)	0
Br14	7807(7)	5000	7739(6)	65.8(17)	113(6)	16.1(9)	68(3)	0	-2(3)	0
Br15	2736(8)	0	7784(7)	75.4(19)	105(6)	15.9(10)	105(5)	0	-1(4)	0
Br16	7755(7)	0	2782(7)	70.3(18)	94(5)	19.8(10)	97(4)	0	5(3)	0

Space Group: *Pm*

a = 11.6324(5) Å, b = 11.7525(6) Å, c = 11.6368(6) Å, β = 89.663(10)°, Dx = 4.842 g/cm^3

Measurement Temperature: 280 K

Crystal Dimensions (diameter): ~50 μm

Wavelength: 0.41328 Å

2θ range for data collection: 2.014° to 37.07°

Index ranges: $-13 \le h \le 16$, $-17 \le k \le 17$, $-17 \le l \le 17$

Reflections collected: 61776

Twin law: 1 0 0 0 -1 0 0 0 -1 2

BASF parameter: 0.136(3)

EXTI extinction parameter: 0.0118(8)

Flack parameter: 0.55(8)

Independent reflections: 10493

Number of fitting parameters: 207

Largest diff. peak/hole: 2.69(Br16)/-4.97(Cs2) e Å^{-3}

R_1 = 5.13 %, wR_2 = 20.92 %, Goodness of Fit = 1.113

*Unit of Atomic displacement parameters is Å^2×10^3.

**The Pseudomerohedry twin fraction components are 0.864(7) and 0.136(3). The Racemic twin fraction components are 0.45(8) and 0.55(8).

Table S9. Structural Parameters from $CsPbBr_3$ at 250 K in *Pm* Space Group

Atoms	x	y	z	U_{eq}	U_{11}	U_{22}	U_{33}	U_{23}	U_{13}	U_{12}
Pb1	2539.9(15)	2501.6(4)	7542.8(10)	23.6(2)	28.1(4)	20.3(3)	22.5(3)	0.01(13)	1.2(2)	1.31(14)
Pb2	7540.9(14)	2501.3(4)	7542.4(10)	23.6(2)	28.0(4)	20.3(3)	22.5(3)	-1.15(13)	1.0(2)	0.03(14)
Pb3	7540.3(14)	2501.9(4)	2542.7(10)	23.6(2)	28.0(4)	20.3(3)	22.6(3)	-0.23(13)	1.0(2)	1.17(14)
Pb4	2541.3(14)	2501.5(4)	2542.8(10)	23.6(2)	28.0(4)	20.3(3)	22.5(3)	-1.38(13)	1.1(2)	0.03(14)
Cs1	9828.2(7)	0	4879(4)	75.5(11)	119(4)	51.1(12)	57.0(18)	0	0.5(17)	0
Cs2	4815.4(7)	0	9854.1(4)	75.3(12)	123(4)	53.5(12)	49.1(15)	0	0.3(16)	0
Cs3	9888.2(6)	0	9736.4(5)	63.6(7)	70(2)	50.8(11)	70.2(17)	0	-6.1(13)	0
Cs4	4894.9(6)	0	4742.3(5)	61.4(7)	69(2)	50.3(11)	64.7(16)	0	-4.1(12)	0
Cs5	5174.8(6)	5000	5189.9(5)	79.1(13)	41.3(14)	46.1(11)	150(4)	0	-6.0(18)	0
Cs6	179.3(6)	5000	239(5)	71.5(10)	43.7(13)	47.2(11)	124(3)	0	-1.2(15)	0
Cs7	5290.9(7)	5000	163.9(5)	63.9(8)	94(2)	44.3(10)	53.7(15)	0	-6.7(13)	0
Cs8	287.2(7)	5000	5161.9(5)	63.7(8)	94(2)	45.2(10)	51.9(14)	0	-4.9(13)	0
Br1	2987.8(6)	2765.3(2)	5052.8(4)	55.5(6)	69.1(16)	80.3(14)	17.2(7)	-2.8(8)	0.3(7)	10.9(11)
Br2	2256.7(7)	5000	7877.3(7)	74.5(15)	142(5)	29.4(13)	51.2(18)	0	-15(2)	0
Br3	7274.1(7)	5000	2877.2(6)	74.0(14)	140(5)	29.3(13)	52.8(19)	0	-13(2)	0
Br4	7989.2(6)	2765.9(2)	50.4(4)	55.0(6)	67.9(16)	79.2(14)	18.0(7)	-3.3(8)	0.1(7)	11.2(11)
Br5	24.4(6)	2773.5(2)	3020.8(4)	53.8(6)	27.4(10)	73.7(14)	60.3(14)	-4.2(10)	4.3(8)	1.6(9)
Br6	7308.8(8)	0	7871.7(6)	71.5(13)	122(4)	25.5(12)	67(2)	0	-7(2)	0
Br7	5035.5(6)	2218.1(2)	2104.2(4)	55.9(6)	24.2(10)	69.6(13)	74.0(17)	-13.1(11)	-1.2(8)	-0.5(9)
Br8	7062.5(6)	2215.9(2)	5059.5(4)	55.1(6)	73.4(17)	67.8(13)	24.3(8)	0.9(8)	2.7(8)	8.8(11)
Br9	2064.9(6)	2218.6(2)	62.1(4)	54.9(6)	73.5(17)	68.3(13)	22.9(8)	1.5(8)	2.8(8)	7.9(11)
Br10	5024.6(6)	2780.2(2)	8018.4(4)	54.8(6)	27.3(10)	75.8(14)	61.4(14)	-9.1(10)	4.3(8)	1.9(9)
Br11	2310.5(8)	0	2873.9(6)	68.7(12)	115(4)	25.6(12)	65(2)	0	-2(2)	0
Br12	33.4(6)	2234.3(2)	7094.1(4)	54.7(6)	24.2(10)	68.4(12)	71.4(16)	-9.2(11)	-1.1(8)	-0.5(9)
Br13	2791.5(8)	5000	2310.5(5)	67.7(12)	59(2)	14.4(9)	130(4)	0	0(2)	0
Br14	7787.7(8)	5000	7302.5(5)	71.1(13)	60(2)	13.4(9)	140(4)	0	-3(2)	0
Br15	2825.1(8)	0	7364.1(5)	59.2(10)	91(3)	11.8(8)	74(2)	0	-0.4(18)	0
Br16	7833.5(8)	0	2374.8(5)	58.4(10)	87(3)	11.0(8)	77(2)	0	-0.9(18)	0

Space Group: *Pm*

a = 11.6205(4) Å, b = 11.7416(5) Å, c = 11.6238(5) Å, β = 89.357(10)°, Dx = 4.857 g/cm^3

Measurement Temperature: 250 K

Crystal Dimensions (diameter): ~50 µm

Wavelength: 0.41328 Å

2θ range for data collection: 2.018° to 37.072°

Index ranges: $-17 \leq h \leq 13$, $-17 \leq k \leq 17$, $-17 \leq l \leq 17$

Reflections collected: 63103

Twin law: 1 0 0 0 -1 0 0 0 -1 2

BASF parameter: 0.136(3)

Flack parameter: 0.57(7)

EXTI extinction parameter: 0.0093 (9)

Independent reflections: 11167

Number of fitting parameters: 207

Largest diff. peak/hole: 7.52(Pb3)/-6.37(Cs7) e Å^{-3}

R_1 = 6.86 %, wR_2 = 25.56 %, Goodness of Fit = 1.120

*Unit of Atomic displacement parameters is Å^2×10^3.

**The Pseudomerohedry twin fraction components are 0.9309(19) and 0.0691(19). The Racemic twin fraction components are 0.43(8) and 0.57(7).

Table S10. Structural Parameters from $CsPbBr_3$ at 120 K in *Pm* Space Group

Atoms	x	y	z	U_{eq}	U_{11}	U_{22}	U_{33}	U_{23}	U_{13}	U_{12}
Pb1	2530.2(17)	2499.7(4)	7530.7(11)	22.0(2)	26.7(4)	18.8(4)	20.1(4)	0.10(15)	1.7(2)	1.21(16)
Pb2	7529.3(16)	2497.9(4)	7529.9(11)	21.9(2)	26.6(4)	18.6(4)	20.4(4)	-1.22(15)	1.7(2)	-0.15(17)
Pb3	7530.9(17)	2499.7(4)	2529.3(11)	22.0(2)	26.6(4)	18.6(4)	20.3(4)	-0.17(15)	1.4(2)	1.10(16)
Pb4	2529.7(16)	2497.8(4)	2530.8(11)	22.0(2)	26.6(4)	18.6(4)	20.4(4)	-1.35(15)	1.6(2)	-0.21(17)
Cs1	10319(7)	0	4867(5)	60.3(8)	73(2)	44.4(11)	55.0(15)	0	-1.9(12)	0
Cs2	5306(7)	0	9867(5)	59.7(8)	74(2)	43.8(11)	53.4(15)	0	0.0(12)	0
Cs3	10187(7)	0	9811(6)	71.0(11)	39.7(15)	47.1(13)	134(4)	0	-8.1(18)	0
Cs4	5194(7)	0	4771(6)	63.6(9)	42.1(15)	47.8(12)	105(3)	0	-1.6(15)	0
Cs5	4869(7)	5000	5345(5)	56.4(7)	61.7(19)	42.0(11)	64.8(18)	0	-0.5(12)	0
Cs6	-123(7)	5000	331(5)	55.1(7)	61.9(19)	41.4(10)	60.0(17)	0	-1.5(12)	0
Cs7	4768(7)	5000	188(5)	64.3(8)	118(3)	40.7(11)	40.7(13)	0	-1.9(15)	0
Cs8	-239(7)	5000	5215(5)	62.0(8)	121(3)	43.1(11)	33.1(11)	0	-1.0(13)	0
Br1	3014(7)	2218(2)	5005(4)	48.1(6)	54.8(14)	67.0(14)	21.7(8)	1.1(8)	2.8(7)	5.2(10)
Br2	2250(8)	5000	7284(7)	63.7(13)	113(4)	12.3(9)	40.6(17)	0	4.3(16)	0
Br3	7255(8)	5000	2271(7)	60.5(12)	108(4)	11.2(9)	43.5(17)	0	4.8(17)	0
Br4	8012(7)	2215(2)	3(4)	48.5(6)	55.4(15)	66.1(14)	22.8(8)	0.0(8)	3.7(8)	7.2(10)
Br5	47(7)	2802(2)	2966(5)	48.7(6)	27.3(12)	60.7(13)	52.3(14)	-10.1(10)	4.4(8)	0.4(9)
Br6	7197(8)	0	7297(7)	62.0(12)	70(3)	9.3(9)	94(3)	0	1(2)	0
Br7	5031(7)	2209(2)	2033(5)	48.0(6)	22.0(11)	62.0(14)	63.3(16)	-4.2(10)	-0.7(8)	0.0(9)
Br8	7065(7)	2797(2)	4989(5)	49.3(6)	70.9(18)	58.4(13)	18.3(7)	-2.1(8)	0.4(8)	10.3(11)
Br9	2062(7)	2801(2)	-8(5)	50.0(6)	72.4(18)	58.9(13)	18.3(7)	-1.7(8)	0.9(8)	9.4(11)
Br10	5045(7)	2783(2)	7974(5)	47.6(6)	27.4(12)	60.2(13)	49.3(13)	-5.0(9)	4.7(8)	1.1(9)
Br11	2207(8)	0	2291(7)	61.2(11)	68(3)	10.4(9)	90(3)	0	3(2)	0
Br12	32(7)	2203(2)	7030(5)	48.6(6)	21.8(11)	63.0(14)	64.1(16)	-9.5(11)	-0.8(8)	0.9(9)
Br13	2834(8)	5000	2798(5)	56.5(12)	74(3)	27.0(13)	71(3)	0	-1(2)	0
Br14	7807(9)	5000	7805(5)	56.6(12)	77(3)	28.5(14)	74(3)	0	-7(2)	0
Br15	2761(8)	0	7861(6)	55.4(11)	85(3)	24.2(13)	83(3)	0	-16(2)	0
Br16	7780(8)	0	2851(6)	54.2(11)	82(3)	23.1(12)	82(3)	0	-15(2)	0

Space Group: *Pm*
a = 11.6096(6) Å, b = 11.7308(6) Å, c = 11.6122(6) Å, β = 89.062(10)°, Dx = 4.871 g/cm^3
Measurement Temperature: 120 K
Crystal Dimensions (diameter): ~50 μm
Wavelength: 0.41328 Å
2θ range for data collection: 2.018° to 37.072°
Index ranges: $-17 \leq h \leq 13$, $-17 \leq k \leq 17$, $-17 \leq l \leq 17$
Reflections collected: 60526
Twin law: 1 0 0 0 -1 0 0 0 -1 2
BASF parameter: 0.0794(17)
Flack parameter: 0.52(7)
EXTI extinction parameter: 0.0109 (11)
Independent reflections: 10996
Number of fitting parameters: 207
Largest diff. peak/hole: 8.05(Pb3)/-5.49(Cs4) e Å^{-3}
R_1 = 7.78 %, wR_2 = 28.60 %, Goodness of Fit = 1.144

*Unit of Atomic displacement parameters is Å^2×10^3.
**The Pseudomerohedry twin fraction components are 0.9206(17) and 0.0794(17). The Racemic twin fraction components are 0.48(7) and 0.52(7).

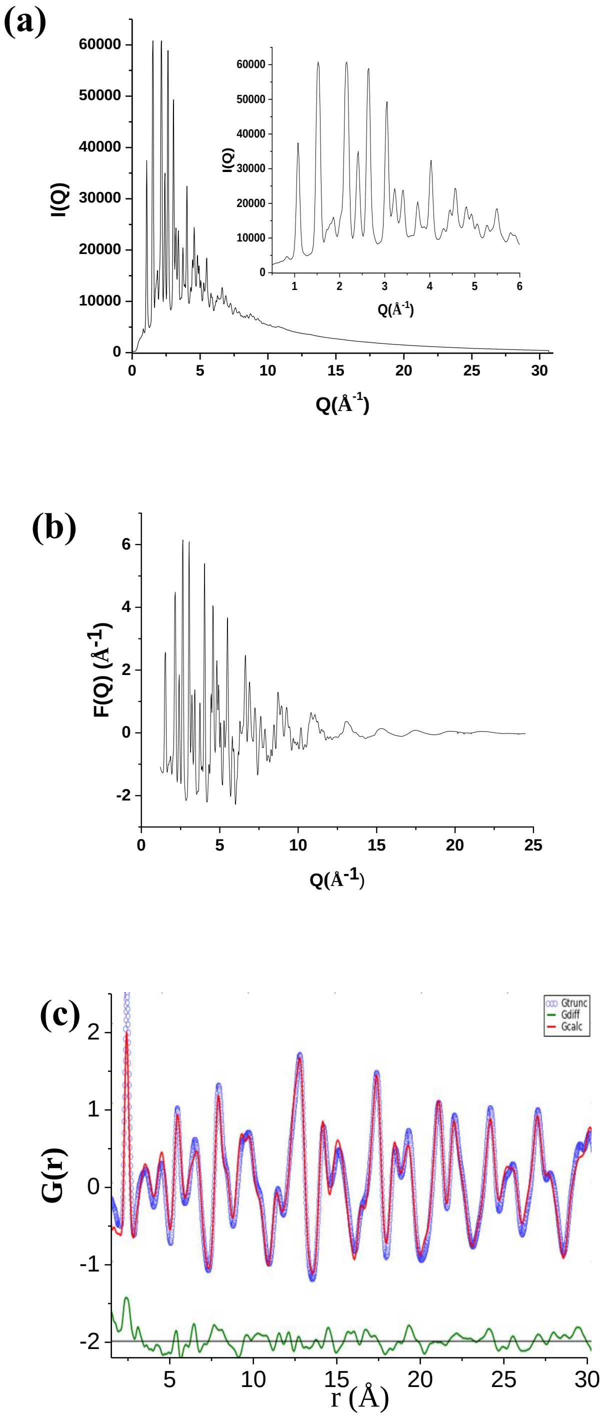

Fig. S14. (a) Raw X-ray scattering data (280 K) without background subtraction (expanded in inset) and (b) reduced scattering data F(Q) at 280 K. A representative fit of the PDF data in real space between 2 Å and 30 Å (c).

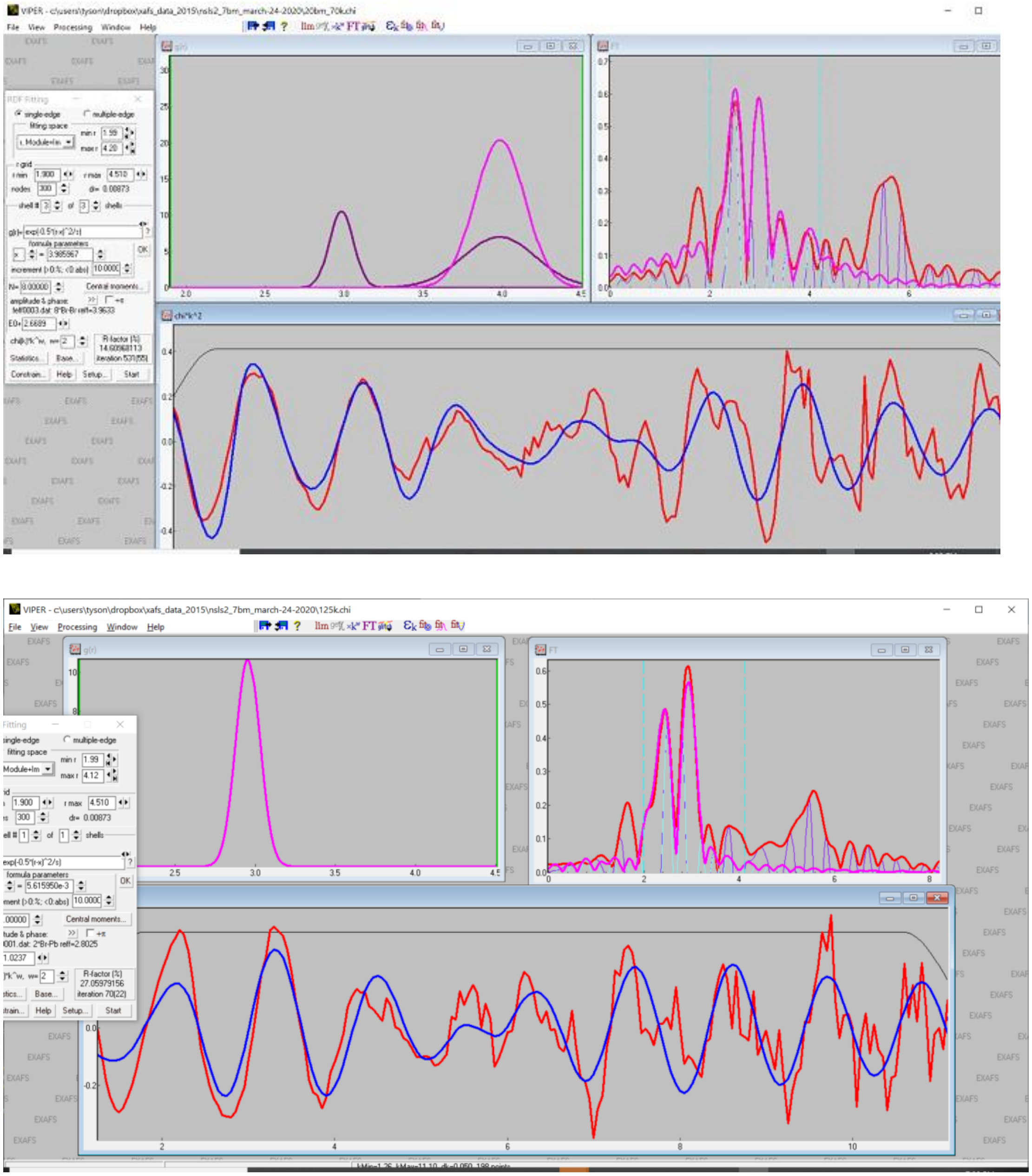

Fig. S15. (Top) Fits of to the Br K-edge x-ray absorption fine structure data at 70 K for the Br-Pb, Br-Cs, and Br-Br shells and (Bottom) at 125 K for a single Br-Pb shell only.

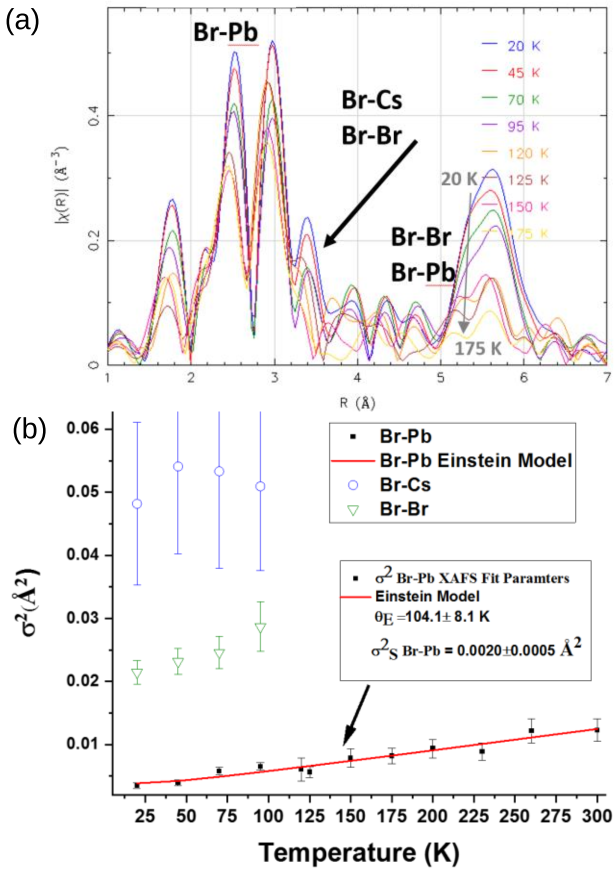

Fig. S16. (a) Fourier transform of XAFS data between 20 and 175 K, indicating suppression of high order peaks beyond Pb-Br above ~170 K. Bold labels indicate the peak assignments (e.g., Br-Pb) and light labels indicate the data temperatures values. Three-shell Fits to XAFS data above ~ 95 K are unstable. (b) Extracted XAFS thermal parameters (σ^2) for the Br-Pb, Br-Cs, and Br-Br atomic pairs for temperatures up to 95 K and for Br-Pb only for higher temperatures. The Br-Pb data was modeled by an Einstein function, yielding a static contribution σ^2_S = 0.0020± 0.0005 Å^2 and Einstein temperature of θ_E = 104.1 ± 8.1 K.

(a) 430 K (Im-3 Z=8)

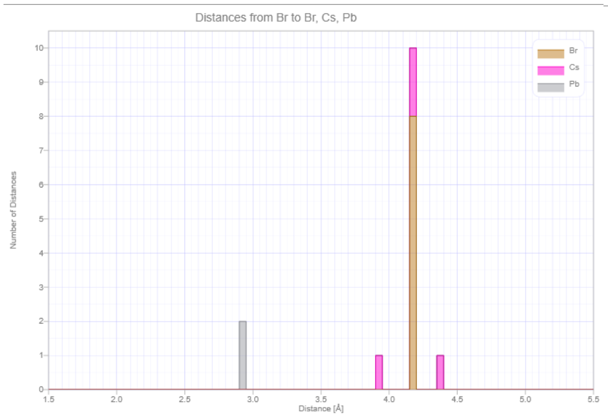

(b) 370 K ($P2_1/m$ Z=8)

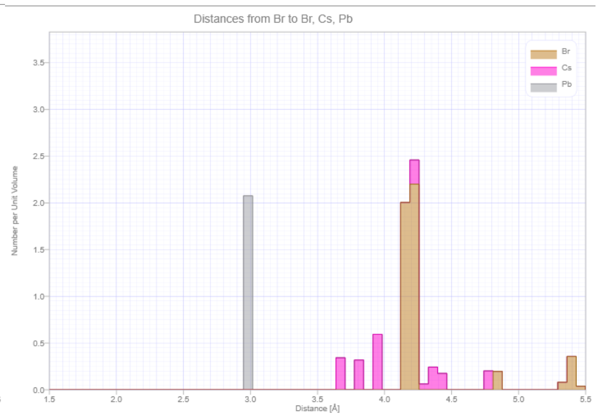

(c) 230 K ($P2_12_12_1$ Z=4)

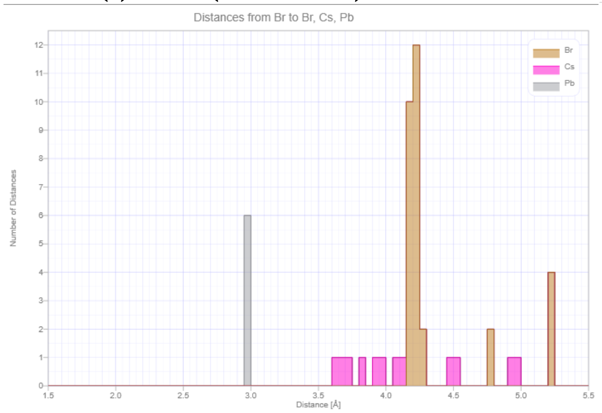

(d) 100 K (($P2_12_12_1$ Z=4)

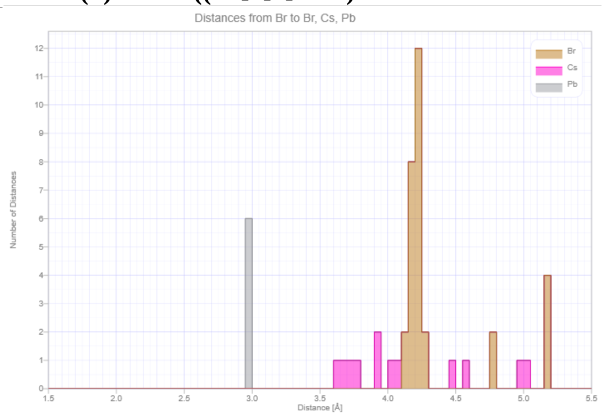

(e) 230 K (Pnma Z=4)

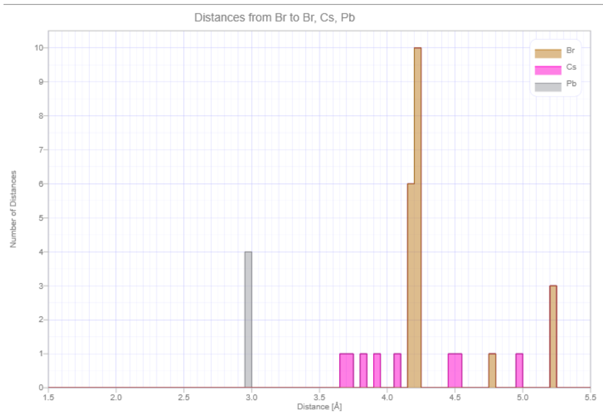

(f) Cubic Structure based on 230 K lattice params. (Pm-3m (Z=1))

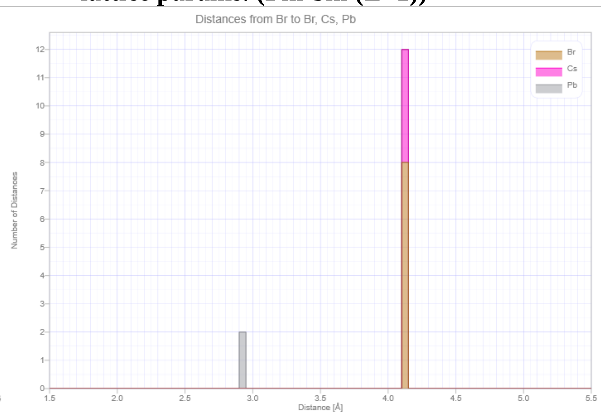

Fig. S17. Atomic pair distributions (number of atom-atom distances vs. r) about Br sites derived from single-crystal data for structural solutions at (a) 430 K, (b) 370 K, (c) 230 K, and (d) 230 K. For completeness, the Pnma structure at 230 K is given in (e), and the approximated distribution for a simple cubic cell based on lattice parameters at 230 K is given in (f). Note that in the real sample (not simple cubic structure), the Cs-Br distribution is never a single peak for temperatures up to 450 K, at least. Note also the large spread in the Br-Cs distribution in the $P2_12_12_1$ space group. This spread in positions becomes less broad in the 100 K structure compared to the 230 K structure.

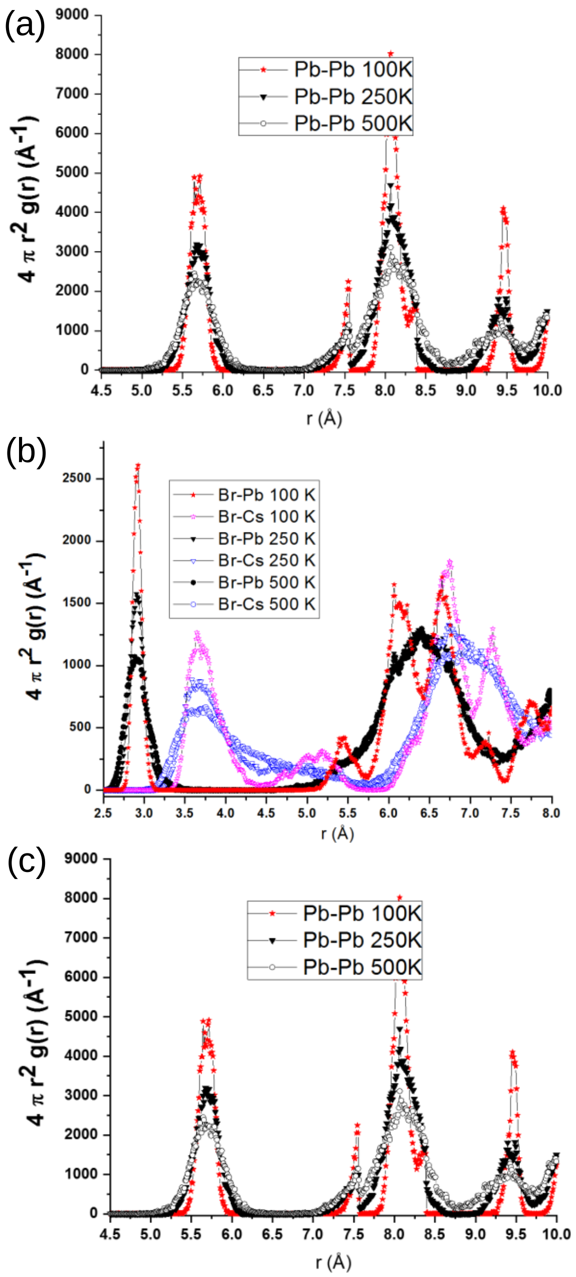

Fig. S18. (a) Radial distribution functions for Pb-Pb pairs at 100, 250, and 500 K derived from ab initio molecular dynamics simulations. (b) Corresponding functions for the Br-Pb and Br-Cs pairs. Note the loss of discrete structure and significant broadening occurring on going from 100 to 250 K compared to the smaller changes in going from 250 to 500 K. In panel (c), note that the same abrupt broadening is seen in the Br-Br pair distributions in going from 100 to 250 K. The results are consistent with significant disordering of the Br-Cs and Br-Pb pairs between 100 and 250 K (for increasing temperature).

References

[1] (a) A. Dewaele A., M Torrent, P. Loubeyre and M. Mezouar Phys. Rev. B **78**, 104102 (2008).
(b) H. K. Mao, J. Xu, and P. M. Bel, J. Geophys. Res. **91**, 4673 (1986).
(c) http://millenia.cars.aps.anl.gov/gsecars/ruby/ruby.htm

[2] Bruker (2016). *APEX3*, *SAINT*, and *SADABS*. Bruker AXS Inc., Madison, Wisconsin, USA.

[3] O. V. Dolomanov, L. J. Bourhis, R. J. Gildea, J. A. C. Howard, and H. Puschmann, J. Appl. Cryst. **42**, 339 (2009).

[4] (a) W. Clegg, Acta Cryst **E75**, 1812 (2019).
(b) D. Watkin, J. Appl. Cryt. **41**, 491 (2008).
(c) R. E. Marsh, Acta Cryst **B51**, 897 (1995).

[5] (a) l. Guzei, R. Herbst-Irmer, A. Munyanezac and J. Darkwad, Acta Cryst **B68**,150 (2012).
(b) S. Pearsons, Acta Cryst. **D59**, 1995 (2003).
(c) R. Herbst-Irmerand G. M. Sheldrick. Acta Cryst B54, 443 (1998).

[6] G. Kresse and D. Joubert, Phys. Rev. B **59**, 1758 (1999).

[7] J. P. Perdew and A. Zunger Rev. B **23**, 5048 (1981).

[8] (a) Atsushi Togo and Isao Tanaka, Scr. Mater., **108,** 1-5 (2015).
(b) L. Chaput, A. Togo, I. Tanaka, and G. Hug, Phys. Rev. B, **84**, 094302 (2011).

[9] (a) T. A. Tyson, W. Gao, Y. S. Chen, S. Ghose and Y. Yan, "Large thermal motion in Halide Perovskites", Sci. Rep. **7** (1) (2017).
(b) T. A. Tyson, T. Wu, H. Y. Chen, J. Bai, K. H. Ahn, K. I. Pandya, S. B. Kim and S. W. Cheong, J. Appl. Phys. **110** , 084116 (2011).

[10] (a) T. A. Tyson, M. Deleon, S. Yoong, and S. W. Cheong, Phys. Rev. B: Condensed Matter and Materials Physics **75**, 174413 (2007).
(b) B. Ravel and M. Newville, J. Synchrotron Rad. **12**, 537 (2005); *X-Ray Absorption: Principles, Applications, Techniques of EXAFS, SEXAFS and XANES*, edited by D. C. Konningsberger and R. Prins (Wiley, New York, 1988).

[11] K.V. Klementev, J. Phys. D **34**, 209 (2001).

[12] T. A. Tyson, M. Deleon, S. Yoong, and S. W. Cheong, Physical Review B **75**, 174413 (2007).

[13] (a) R. B. Neder and Th. Proffen, Diffuse Scattering and Defect Structure Simulations, (Oxford University, Oxford, 2008).
(b) T. Egami and S. L. J. Billinge, Underneath the Bragg Peaks: Structural Analysis of Complex Materials, (Pergamon, Amsterdam, 2003).
(c) Th. Proffen, S. J. L. Billinge, T. Egami and D. Louca, Z. Kristallogr **218**, 132 (2003).
(d) V. Petkov, in Characterization of Materials, (John Wiley and Sons, Hoboken, 2012).
(e) C. L. Farrow, P. Juh´as, J. W. Liu, D. Bryndis, E. S. Boˇzin, J. Bloch, Th. Proffen and S. J. L. Billinge, J. Phys.: Condens. Matter, 19, 335219 (2007).

(f) P. Juhás and T. Davis, C. L. Farrow, S. J. L. Billinge, J. Appl. Cryst. **46**, 560-566 (2013).
(g) Extraction of the scattering data from the 2D detector was conducted using FIT2D http://www.esrf.eu/computing/scientific/FIT2D/

[14] R.E. Whitfield, D. J. Goossen, and T. Richard Welberry, IUCrj **3**, 20 (2016).

[15] C. Prescher and V. B. Prakapenka, *High Pressure Res*. **35**, 223 (2015).
